# Assembling Lipid Membrane Scaffolds on Microgel-Based Artificial Cells through Vesicle Fusion onto the Hydrogel Network

**DOI:** 10.1021/acsnano.5c12532

**Published:** 2026-01-08

**Authors:** Matthew E. Allen, James W. Hindley, Maya I. Müller, Kexin Cai, Marina K. Kuimova, Robert V. Law, Simon D. Connell, Seraphine V. Wegner, Oscar Ces, Yuval Elani

**Affiliations:** † Department of Chemistry, Molecular Sciences Research Hub, 4615 Imperial College London, 82 Wood Lane, London W12 0BZ, U.K.; ‡ Institute of Chemical Biology, Molecular Sciences Research Hub, Imperial College London, 82 Wood Lane, London W12 0BZ, U.K.; § Department of Chemical Engineering, Exhibition Road, Imperial College London, South Kensington, London SW7 2AZ, U.K.; ∥ FabriCELL, Molecular Sciences Research Hub, Imperial College London, 82 Wood Lane, London W12 0BZ, U.K.; ⊥ Institute of Physiological Chemistry and Pathobiochemistry, 9185 University of Münster, Waldeyerstraße 15, 48149 Münster, Germany; # School of Physics and Astronomy, 4468 University of Leeds, Leeds LS2 9JT, U.K.

**Keywords:** hydrogels, lipid membranes, artificial cells, biomimicry, self-assembly

## Abstract

Artificial cells assembled from materials such as hydrogels have emerged as platforms to replicate and understand biological functionalities, processes, and behaviors. However, hydrogels lack a lipid membrane, a vital property of cellular systems. Here we develop a process for the assembly of a fluid and stable lipid membrane which coats the hydrogel mesh network within the particle, through electostatically-mediated fusion of nanoscale lipid vesicles. This confers cell-mimetic and biotechnologically relevant properties upon microscale, cell sized, hydrogel artificial cells generated through microfluidics. We exploit the properties of the created membrane to augment existing hydrogel properties through permeability alteration and protection of the hydrogel from small molecule degraders. Furthermore, we show that the lipid membrane is compatible with organelle substructures within the hydrogels, which enables the exploitation of an enhanced material design space to build hydrogel artificial cells that increasingly mimic the organization of cells. This platform paves the way for producing next generation artificial cells and functional microdevices from interfaced hydrogel-lipid materials. Our technologies may underpin new opportunities for integrating membranes into hydrogel-based systems, inlcuding for drug delivery and tissue engineering.

## Graphical abstract

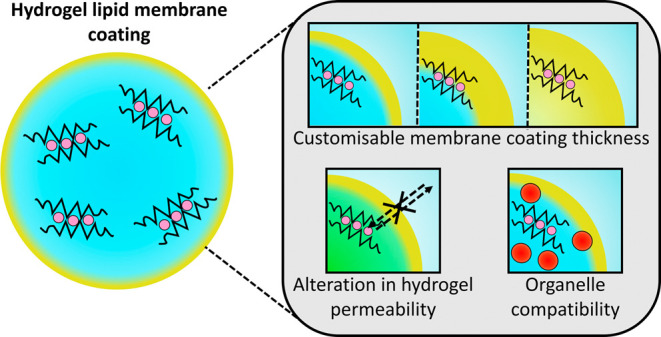

## Introduction

Artificial cells have been developed as tools that can reproduce the form, function, and behaviors of living biological cells.^1–4^ Cellular features that have been successfully mimicked include compartmentalization,^5^ motility,^6^ communication,^7^ signal transduction^8,9^ and growth.^10^ In order to achieve replication of these biological behaviors, artificial cells have been assembled from diverse materials including lipids,^11^ coacervates,^12,13^ polymers^14^ (including DNA^15^) and hydrogels.^16^ Through utilizing these materials, artificial cells have enormous capabilities as next generation biotechnological devices for applications which include drug delivery,^17^ biosensing^18^ and as microreactors.^19^

Of these materials, hydrogels have emerged as an excellent chassis for the formation of artificial cells^20^ due to their ability to replicate crowded cellular environments such as the cytoplasm^21^ and the extracellular matrix^22^ through possessing a 3D interconnected molecular network that contains a large volume of water^23^ and is porous to small molecules.^24^ Hydrogels are also biocompatible,^25,26^ have adjustable mechanical properties^27,28^ and can be engineered to have functionality across a large chemical space.^29,30^ Furthermore, hydrogels can incorporate a wide range of functional organelles^31^ and have a potential role in the origin of life.^32^ All of these properties are useful in the construction of complex biomimetic cell mimics.

However, hydrogels lack a fluid lipid membrane, a fundamental feature that cells possess that serves multiple roles, critically compartmentalization^33^ and controlling permeability of low molecular weight (*M*_W_) species.^34^ Hence hydrogels struggle to recreate dynamic membrane processes including compositional remodelling,^35^ shape deformations^36^ and domain organization^37^ in addition to not containing important biological molecular machines such as membrane proteins,^38^ which underpin many fundamental cellular phenomena. Consequently, effective integration of lipid membranes and hydrogels is critical for the advancement of hydrogels as artificial cells. In current examples of lipid membrane enclosed hydrogel artificial cells^39–48^ (or liposomes embedded within hydrogels)^49–52^ only basic functionality has been demonstrated. An expansion of this functional design space is required to unlock the full potential of lipid‑membrane‑coated hydrogels, for instance, through the development of alternate architectures which can mimic lipid rich cellular structures such as the Golgi apparatus.^53^

Herein, we solve this problem by designing and developing a straightforward, versatile and programmable strategy for applying a fluid and stable membranous coating to microfluidic generated micron sized hydrogel based artificial cells that directly assembles on the internal hydrogel structure through the electrostatically-mediated fusion of nanoscale lipid vesicles (Figure 1). In comparison to previously developed platforms, our membranous coating possesses a unique architecture where the membrane can cover the entire hydrogel network (i.e. directly coats the hydrogel mesh at the core of the particle), instead of being supported around the outside of the hydrogel^39–48^ or embedded as liposomes within the hydrogel.^49–52^ The membranous coating can be assembled onto alginate hydrogels without the need for further chemical modification of the hydrogel^41^ or complex microfluidic strategies^39,44^ which other systems suffer from, and uniquely, the penetration depth of the membrane coating can be readily adjusted by altering the vesicle incubation period. The differences between our membranous coating and the literature are further detailed in the Supporting Information (Table S1). Our alternative membrane architecture possesses properties mimicking a biological lipid membrane, and the membrane is compatible with the inclusion of vesicular organelles in the hydrogel. The functional role of the membrane coating is demonstrated by its ability to enhance hydrogel artificial cells through controlled modulation of permeability to both hydrophobic and hydrophilic small molecules. Our platform may enable the creation of advanced artificial cells and lipid-hydrogel microdevices, while opening new directions for soft matter systems in fields ranging from drug delivery, bioelectronics and tissue engineering.

**Figure 1:**
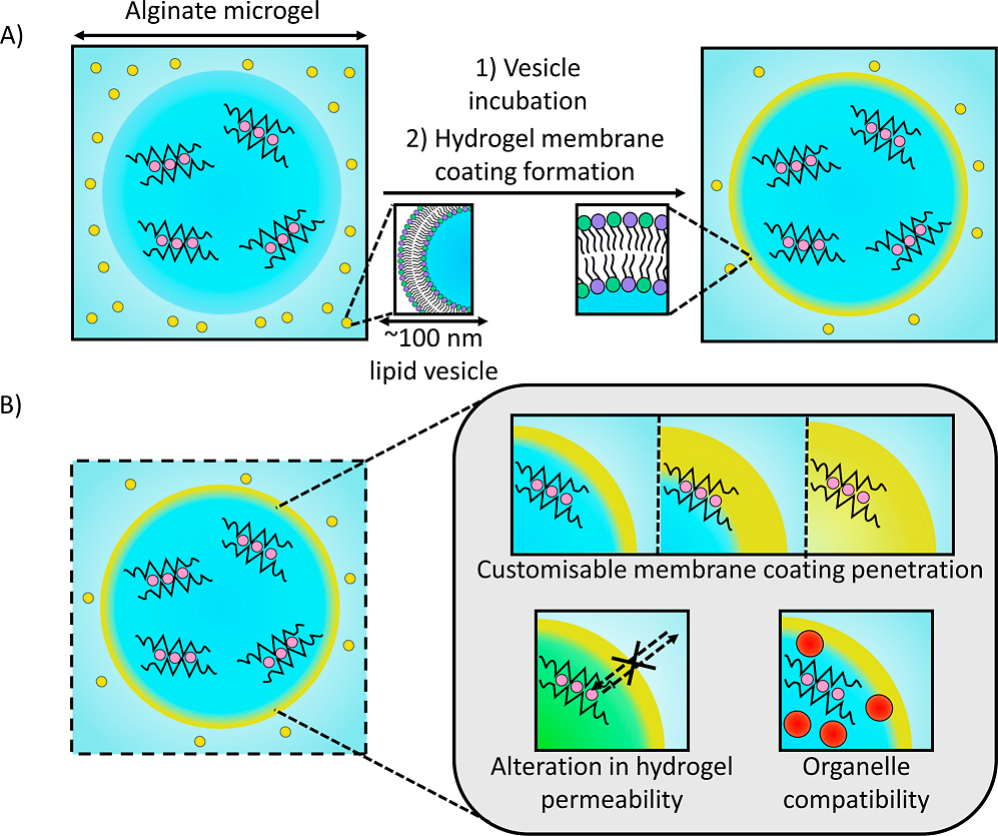
Overview of the process and properties gained from membrane assembly on hydrogel artificial cells. (A) Diagram of the hydrogel artificial cell membrane coating formation process. This occurs through lipid vesicle incubation, which leads to the formation of a membrane coating on the artificial cells. Diameters of the microgels and liposomes are ∼100 μm and ∼100 nm respectively. (B) Illustration of the properties and functionality the membrane coating imparts on hydrogel artificial cells. These include alteration of the membrane coating penetration depth, alteration in the permeability of the hydrogel and the compatibility of the membrane coating with internal vesicular organelles.

## Results and Discussion

### Production and Assembly of Hydrogel Artificial Cell Membranes

Initially we produced hydrogel artificial cells which were based on ∼100 μm alginate microgel particles without a membrane coating through the use of a microfluidic device (Figures 2A, S1 and S2 and Video S1) following a previously developed procedure,^54^ where aqueous droplets containing alginate and a calcium-ethylenediaminetetraacetic acid (Ca-EDTA) complex were formed in a continuous oil phase. The droplets containing the hydrogel precursors then meet a second oil phase that contains acetic acid. The acetic acid causes dissociation of the Ca^2+^ from the EDTA enabling the Ca^2+^ to bind to the alginate, thus forming micron scale hydrogel beads. The hydrogels were then collected and resuspended in aqueous buffer. A membrane coating was then created around the hydrogels by adding small unilamellar vesicles (SUVs; 120 nm diameter) (Figure S3) to the hydrogel containing solution. In solution with the hydrogels, the total lipid concentration was larger than the critical micelle concentration,^55^ thus the lipids existed and interacted with the hydrogels predominantly as lipid vesicles and not as single amphiphiles, limiting diffusion of single lipid amphiphiles into the hydrogel structure. The vesicles were attracted to the hydrogels through electrostatic interactions (Table S2) whereby they assembled into a membranous coating on the hydrogel surface. This strategy of vesicle electrostatic attraction has been used previously to adhere SUVs to artificial cell membranes.^56^ Upon incubation with vesicles having a lipid composition containing 1,2-dioleoyl-3-trimethylammonium-propane (chloride salt) (DOTAP) and 1,2-dioleoyl-*sn*-glycero-3-phosphoethanolamine (DOPE), fluorescence localization could be seen around the hydrogels (Figures 2B and S4), this localization was present around the entire gel (Figure 2C) indicating that the vesicles were forming a membrane structure around the hydrogels.

**Figure 2:**
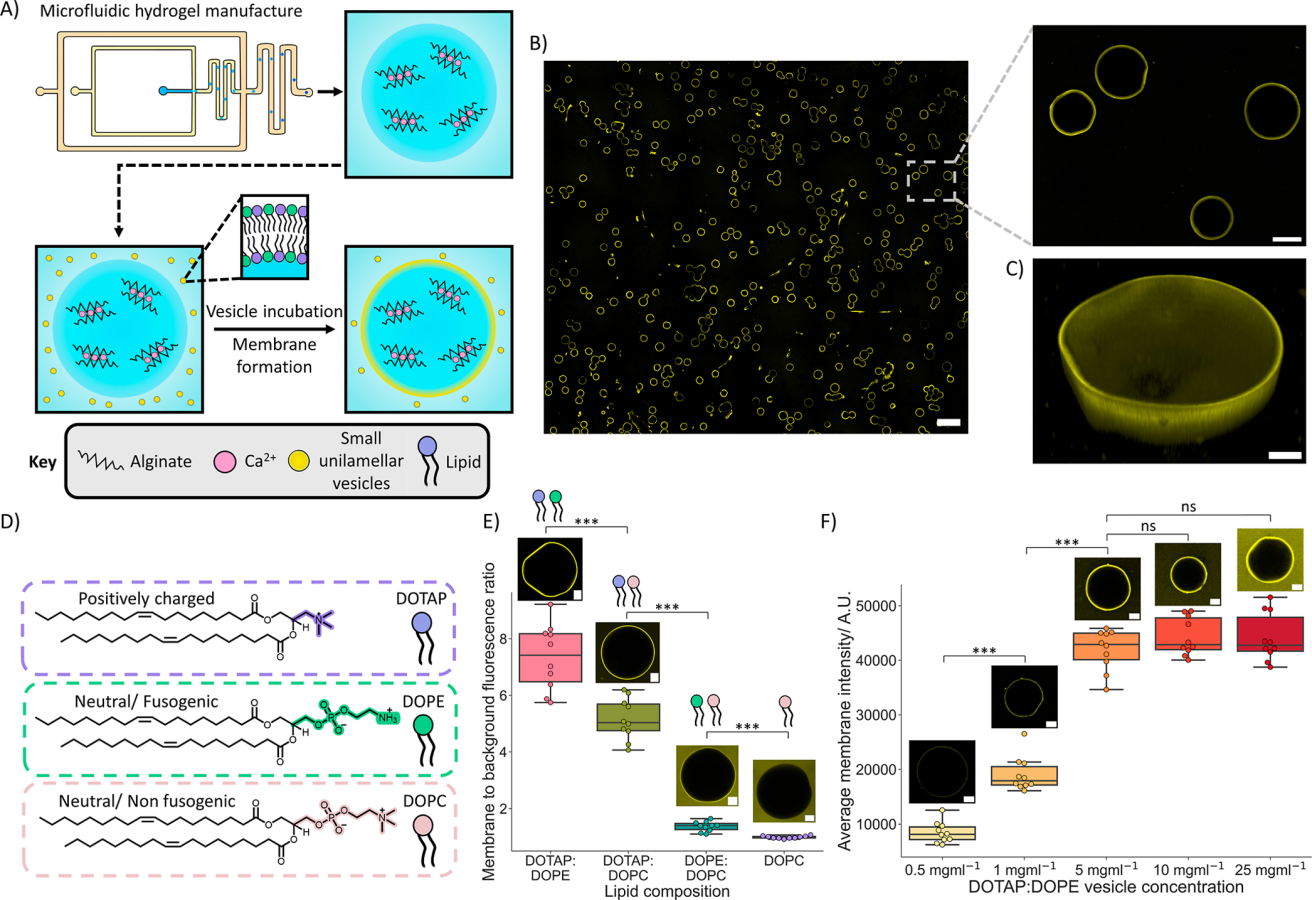
Assembly of a membrane on hydrogel artificial cells. (A) Schematic illustrating the processes to create and form a membrane on the hydrogel artificial cells. The hydrogel artificial cells are produced using a microfluidic chip before being incubated with a population of small unilamellar vesicles which form the membrane. (B) A tiled fluorescence confocal image of a population of hydrogel artificial cells possessing a membrane comprised of a DOTAP:DOPE lipid composition with a rhodamine tag, the boxed region is enlarged in the related image. The scale bar on the tiled image is 250 μm and scale bar on the enlarged image is 50 μm. (C) A 3D confocal Z stack of the membrane surrounding a hydrogel artificial cell, the membrane can be seen to uniformly encompass the entire hydrogel. The scale bar is 20 μm. (D) Diagrams and structures of the different lipids used to form the vesicle compositions to coat the hydrogel artificial cells. (E) A box plot with accompanying representative fluorescence confocal images demonstrating how lipid vesicle composition impacts the ability to form a membrane on the hydrogel artificial cells. Upon the usage of both positively charged (DOTAP) and more fusogenic lipids (DOPE) than DOPC, an increase in signal on the hydrogel is seen, due to increased electrostatic interactions between the lipid vesicles and the negatively charged alginate and increased fusogenicity of the lipid vesicles. The scale bars are 20 μm. (F) A box plot with accompanying representative fluorescence confocal images demonstrating how DOTAP:DOPE concentration impacts upon the intensity of the formed membrane on the hydrogel artificial cells. Increasing the lipid concentration up to 5 mg mL^–1^ results in a more prominent membrane. After 5 mg mL^–1^ the membrane intensity remains constant with increasing lipid concentration. The scale bars are 20 μm. All of the box plots were produced from analyzing *n* = 10 hydrogel artificial cells for each condition.

To investigate how this assembly process was occurring we incubated the hydrogels in a variety of vesicles with different lipid compositions (Figures 2D,E and S5). It could be seen that when using a lipid composition with a neutral charge and which forms stable lipid bilayers (1,2-dioleoyl-*sn*-glycero-3-phosphocholine (DOPC)),^57^ no fluorescent coating on the hydrogels was observed. The vesicles were also excluded from the hydrogel interior due to the pore size of the alginate hydrogel (∼5 nm)^58^ being significantly smaller than the vesicles. Upon introducing a positively charged lipid (DOTAP) into the vesicle composition in a 1:1 molar ratio, significant localization on the hydrogel surface was observed, this is due to alginate possessing a negative charge^59^ so the vesicles were attracted electrostatically to the hydrogel surface, causing localization. Upon replacing DOPC with DOPE, a neutral lipid that is heavily used in fusogenic compositions due to its high curvature^60^ that arises from the lipid headgroup occupying significantly less space than the tailgroup, another increase in membrane localization was observed, suggesting that the DOPE lipid was promoting membrane assembly on the hydrogel surface. Furthermore, a minor increase in localization was seen in an entirely neutral lipid composition (DOPE:DOPC) compared to DOPC confirming that the addition of DOPE is enhancing membrane assembly on the hydrogel surface, however this change was not as significant as the change produced from the addition of DOTAP. These results demonstrate that by tuning the lipid composition the assembly of vesicles on a hydrogel surface can be altered and combining charge attraction (DOTAP) with fusogenicity (DOPE) appears to maximize membrane assembly on the hydrogel surface. Equimolar compositions were selected of DOTAP:DOPC and DOTAP:DOPE vesicles due to their previous uses as cationic^61^ and fusogenic^62^ compositions, respectively.

To further demonstrate the tuneability of the membrane assembly process, we also added different concentrations of DOTAP:DOPE vesicles to solutions containing hydrogel artificial cells (Figure 2F). On increasing the concentration of DOTAP:DOPE vesicles up to 5 mg mL^–1^, the higher the membrane intensity on the hydrogel artificial cells. Above 5 mg mL^–1^ the intensity of membrane on the hydrogel artificial cells remained constant, however the intensity of the surrounding vesicles increased (Figure S6), indicating that the hydrogel artificial cell interface is saturated with vesicles at 5 mg mL^–1^ and highlighting that this concentration is an ideal concentration to use. The results show that the prominence of the membrane coating is vesicle concentration dependent. Through showing the ability to deposit membranes on the hydrogel artificial cells of a tunable prominence and with a variety of lipid compositions, we enable our system to mimic a host of membrane-associated processes and behaviors that rely on lipid diversity.

On leaving the hydrogel artificial cells in the DOTAP:DOPE vesicle solution, we also observed that the membrane coating began to penetrate into the hydrogel (Figure 3A,B) (Videos S2 and S3) and alter the optical texture (Figure S7). This effect was not observed in DOTAP:DOPC vesicles which also coated the hydrogel artificial cells (Figure S8). To investigate this further we monitored the hydrogels immersed in vesicle solution for 8 h (Figure 3B,C) (Figure S9). From the extracted line profiles, we could see an increase in distance the membrane covers on the hydrogel and the intensity of the membrane, demonstrating that upon fusion of the DOTAP:DOPE vesicles on the hydrogel surface, the vesicles coat the hydrogel network and will form a membrane structure which penetrates into and eventually covers the entire hydrogel structure. Further analysis of the hydrogel coating process revealed an overall increase in the fluorescence signal on the hydrogel artificial cells (Figure 3D) and a slight decrease in hydrogel area (Figure 3E) The slight decrease in area can be attributed to the lipid membrane reducing the strength of interactions between the alginate structure and the aqueous solution^63^ while the increase in fluorescence occurred due to an increasing amount of lipid being deposited on the hydrogel artificial cells over time. Both processes had similar kinetic profiles, suggesting that the decrease in hydrogel area is linked to the increase in lipid present on the hydrogel structure. By removing the external vesicle solution from the partially coated hydrogel artificial cells, the membrane coating process was stopped and the partially coated membrane on the hydrogels remained the same size (Figure S10), enabling the generation of hydrogel artificial cells with stable coatings of defined penetration depths. We also observed that the DOTAP: DOPE vesicles only formed a prominent penetrative membrane coating at DOTAP concentrations of 50 mol % or higher (Figure S11). Thus, demonstrating that a balance between positive charge and degree of fusogenicity is important for forming these penetrative coatings.

**Figure 3:**
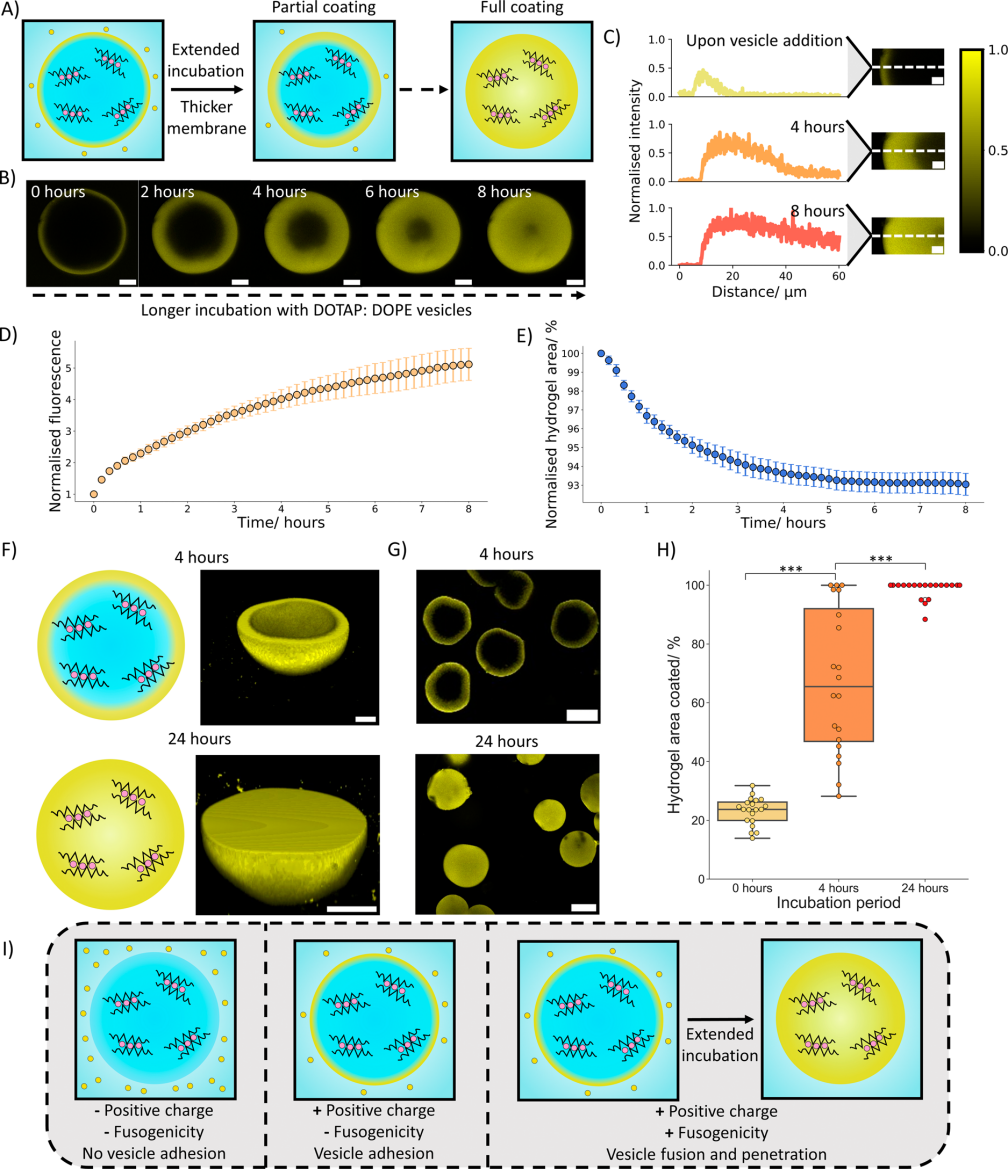
Formation of penetrative membrane coatings on hydrogel artificial cells. (A) Schematic showing that upon longer DOTAP:DOPE vesicle incubation times, the membrane upon the hydrogel artificial cells increases in size until it covers the entire hydrogel structure. (B) Corresponding confocal microscopy images showing the increase in membrane coating size over 8 h. The scale bars are 20 μm. (C) Line profile graphs with corresponding confocal microscopy images demonstrating how the membrane penetrates further into the hydrogel over 8 h. The dotted lines on the images represent the line profiles taken for the graphs. Over the 8 h it can be seen that the membrane coating penetration depth increases and coats the entire hydrogel section. The density of the membrane also increases, shown by the increase in fluorescence intensity, until the gel network is saturated with membrane, at which point the intensity remains constant. The scale bars are 10 μm. (D) A graph showing how the fluorescent signal on the hydrogel artificial cells changes over 8 h of membrane coating. (E) A graph illustrating how the area of the hydrogel artificial cells changes over 8 h of membrane coating. Graphs (D,E) are produced from *n* = 10 hydrogel artificial cells and the error bars are the standard deviation of the population. (F) Illustrations and 3D Z stack projections of hydrogels coated with vesicles for 4 and 24 h. The scale bars are 20 μm. (G) Population images of hydrogel artificial cells with 4 and 24 h coatings applied. The scale bars are 50 μm. (H) A box plot showing how the vesicle incubation time affects the membrane coating coverage on the hydrogel artificial cells. A longer incubation time leads to larger coating coverage. *n* = 20 hydrogel artificial cells were analyzed. (I) A diagram showing how vesicle charge and fusogenicity impact membrane formation on the hydrogel artificial cells. Vesicles with a positive charge and a degree of fusogenicity are required to form a penetrative membranous coating.

Further analysis on populations of hydrogel artificial cells with partially penetrated (4 h vesicle incubation) or fully penetrated (24 h vesicle incubation) membrane coatings (Figure 3F,G) revealed that the membranous coating could be seen to be homogeneous across the hydrogel (Figure S12) and the optical texture of the hydrogel had drastically darkened (Figure S13). On a population level (Figure 3H), after 4 h of vesicle incubation, almost all of the analyzed hydrogel artificial cells possessed an increased area coated than after no incubation. However, the distribution was broad, indicating a range of coating penetration depths were present. This aligns with the variation in coating sizes seen in the coating kinetic traces after 4 h (Figure S9). The range of penetration depths may be attributed to the hydrogels having different surface area to volume ratios due to their different sizes and shapes. More vesicles will be able to interact with hydrogels with larger surface areas, thus increasing the speed of coating and proportion of the hydrogel coated on hydrogels with larger surface area to volume ratios. At 24 h, a fully penetrated membrane coating could be seen on almost all of the hydrogels in the population, showing that all hydrogels will eventually possess a full membrane coating if left for long enough in a solution of DOTAP:DOPE vesicles. Fully penetrated membrane coatings could also be seen to have a lipid concentration significantly larger than the vesicle solution used to produce them and the lipid concentration was dependent on the wt % of alginate used to create the hydrogel artificial cells (Figure S14).

By showing that DOTAP:DOPE membranes of tunable penetration depths may be deposited on the hydrogel artificial cells, we unlock the ability to begin to mimic more membrane rich cellular structures such as the Golgi apparatus^53^ and endoplasmic reticulum^64^ using our membranous hydrogel artificial cells. Consequently, increasing the utility of the hydrogel artificial cells. The lipid vesicle composition used to form the penetrative coatings possessed a degree of charge (DOTAP) and fusogenicity (DOPE) (Figure 3I). Furthermore, the DOTAP:DOPE lipid composition used to produce the membrane coating has been extensively used in successful biocompatible lipid nanoparticle formulations for mRNA/gene delivery^65,66^ despite a degree of cytotoxicity shown by DOTAP lipids.^67,68^ To confirm the biocompatibility of the membranous hydrogel artificial cells, we cocultured the artificial cells overnight with HEK 293 cells and performed a viability assay (Figure S15). We observed that the cells were viable after coculture, thereby indicating that the membrane coating possesses a degree of biocompatibility. However, future efforts could investigate reducing the amount of DOTAP present in the lipid composition or utilizing a different, less cytotoxic cationic lipid, for instance DOTMA, to further increase the biocompatibility.^69^

### Characterization of Hydrogel Artificial Cell Membranes

After demonstrating the range of membranous structures DOTAP:DOPE vesicles could form on hydrogel artificial cells, we then investigated the properties of the membranous coating. To begin with we performed fluorescence recovery after photobleaching (FRAP) experiments on newly created membranes (Figure 4A,B) (Video S4). We observed that after photobleaching there was fluorescence recovery of the membrane, suggesting that the membrane was contiguous and not a collection of adhered vesicles on the hydrogel. As a comparison, we performed the same experiment on hydrogels with a DOTAP:DOPC membrane (Figure S16) (Video S5) and saw little membrane recovery indicating that the inclusion of DOPE was facilitating fusion between the vesicles and onto the hydrogel surface and not adhesion of the vesicles to the hydrogel surface. We then additionally encapsulated 4 kDa FITC-dextran into both of the vesicle compositions (Figure S17). Upon vesicle addition to the hydrogels, the vesicle composition containing DOPC produced a faint ring around the hydrogels, again suggesting vesicle adhesion while the DOPE composition had no ring, indicating fusion of the lipid vesicles into a contiguous structure on the hydrogel surface. Furthermore, as the individual vesicles were larger than the pore size of the hydrogel, upon fusion onto the hydrogel network the vesicles were deforming into a smaller bilayer-based structure. This was further supported by an experiment (Figure S18) showing that the membranous coating can be comprised of multiple DOTAP:DOPE vesicle populations, with the fluorescent signal from the different vesicle populations spread evenly throughout the membrane, indicating that the different vesicles are fusing together on the hydrogel to form the membranous coating. Additional photobleaching experiments on hydrogel artificial cells with membrane coatings that penetrate into the hydrogel (Figures S19 and S20) (Video S6) saw that the membrane possessed the same fluidity throughout the penetrated membrane, again indicating that the vesicles were producing an interconnected fluid membrane upon the hydrogel network.

**Figure 4:**
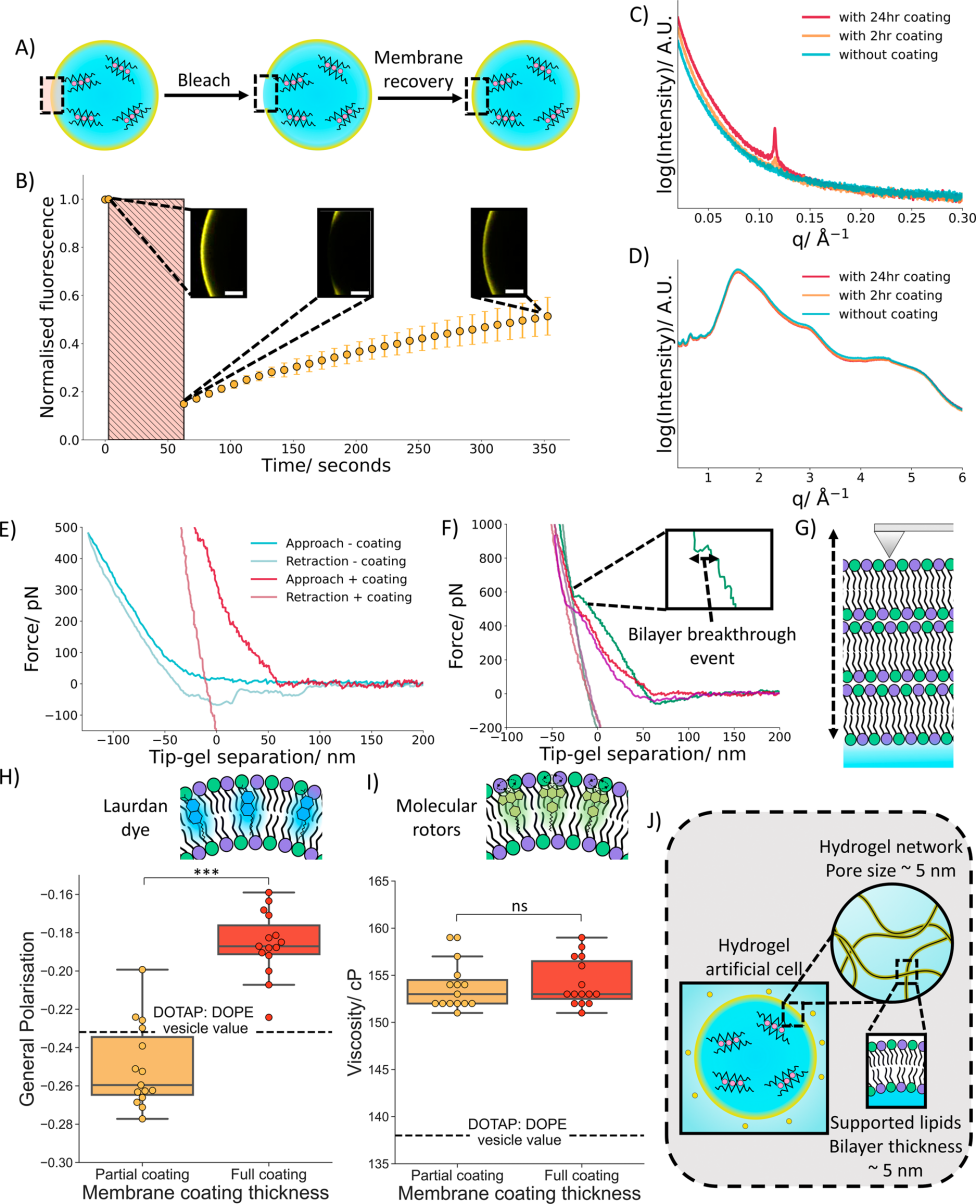
Structure and properties of the membrane on hydrogel artificial cells. (A) A diagram showing recovery of the lipid membrane upon photobleaching a region. (B) Corresponding fluorescence recovery after photobleaching graph with embedded confocal microscopy images showing the recovery of the lipid membrane upon bleaching. This indicates that the membrane is fluid and not a collection of adhered vesicles. (C) A small angle scattering plot showing that upon coating alginate hydrogels with DOTAP:DOPE vesicles, a diffraction peak is seen. With longer incubation periods the more prominent the peak becomes. (D) A wide angle scatting graph demonstrating that upon coating application no change in fluidity is seen. This further confirms that the coating has fluid like characteristics. (E) An AFM force separation curve taken with the same probe on a hydrogel artificial cell with and without a membranous coating. The bare hydrogel artificial cell has a soft, deformable nature and some hysteresis and adhesion is evident upon retraction with the gel surface not instantaneously recovering at the tip velocity used (1.0 μm s^–1^). Meanwhile the coated hydrogel artificial cell presented a different curve, indicating a change in properties upon application of the membrane coating. (F) 3 AFM force separation curves of a hydrogel artificial cell with a membranous coating, the lighter regions indicate the retraction portion of the curve. The curves show a characteristic final penetration of around 600 pN with between 7 and 14 bilayer breakthrough events, shown by the sudden change in curve direction, one of these events is zoomed into and highlighted by an arrow. (G) A schematic of an AFM tip perturbing the stacked lipid bilayers present on the hydrogel artificial cell surface. (H) A box plot contrasting the degree of general polarization as measured by Laurdan dye within coated hydrogel artificial cells membranes, and the polarization of the dye in free DOTAP:DOPE vesicles shown by the dotted line. The coated hydrogel artificial cells show a small deviation from the bulk vesicle general polarization value. (I) A box plot comparing the viscosity of coated hydrogel artificial cells membranes measured by fluorescence lifetime of BODIPY-rotor dye; the viscosity of free DOTAP:DOPE vesicles are shown by the dotted line. The coated hydrogel artificial cells show a small increase in viscosity from the bulk vesicles. For both box plots *n* = 15 hydrogel artificial cells were analyzed for each condition. (J) Schematic detailing the proposed membrane structure on the hydrogel artificial cells when coated with DOTAP:DOPE vesicles. Upon incubation the vesicles form a lipid bilayer supported on the hydrogel network.

To showcase the applicability of the formed membrane and to further support the formation of a membrane across the entire hydrogel network we incubated a millimeter sized hydrogel (∼20 times larger than the hydrogel artificial cells) in the same DOTAP: DOPE lipid vesicle solution (Figure S21). Once again, a membrane was evident demonstrating that this technique to create a membrane on alginate hydrogels can be applied across a range of length scales, a vital tool for integrating lipid membranes into the field of tissue engineering and on hydrogel based biodevices. Moreover, we performed small and wide-angle X-ray scattering experiments on the larger membrane coated hydrogels and observed the appearance of a sharp peak upon membrane assembly in the small angle spectra (Figure 4C), this sharp peak corresponds to a lipid structure being deposited upon the hydrogels,^70^ again supporting the formation of a membrane that penetrates into the hydrogels. With longer incubation periods the more prominent the peak becomes this represents the coating covering more of the hydrogel as there is more membrane to scatter the X-rays. The sharp diffraction peak also further supports that the membrane coating is homogeneous, an inhomogeneous coating would have multiple regions with different membrane structures, which would lead to broadening of the diffraction peak. The absence of additional peaks could be due to the weak signal of the primary peak. Meanwhile the wide angle scattering spectra (Figure 4D) remained the same upon membrane assembly, possessing broad diffraction peaks, indicating that the membrane was fluid,^71^ thus supporting the previous photobleaching experiments.

To further quantify the properties of the formed membranous coating, atomic force microscopy (AFM) was carried out on hydrogel artificial cells with and without the DOTAP:DOPE membrane coating (Figure 4E,F). Without the membrane coating the hydrogel artificial cells had a smooth force separation curve. On coating, the force separation curve changed and now exhibited several sharp changes in direction, consistent with the AFM probe tip rupturing multiple lipid bilayers.^72^ This matched the appearance of results obtained on a solid mica surface (Figure S22), a surface previously used to analyze DOTAP:DOPE bilayers.^73^ The curves showed a characteristic final penetration of around 600 pN with typically between 7 and 14 bilayer breakthrough events but a maximum of approximately 50 bilayers was observed. The large number of bilayers blurred out the individual bilayer penetration events (Figure S23) and a large adhesion force (out of range of graph [Fig fig4]F) was present due to pulling out of the multiple bilayers. It was also observed that the overlay between the approach and retraction curves was not nanometre perfect (as seen on mica (Figure S22)) because the local surface elastic modulus varied, leading to differences in surface deformation. Therefore, AFM again confirmed that a membranous coating was present on the hydrogel surface and revealed that the membranous coating had a multilamellar structure (Figure 4G). Further analysis indicated that the individual bilayers of the membranous coating possessed a bilayer thickness on the order of a cell membrane (Supporting Note S1).

The characteristics of the formed membranous coatings were additionally investigated with environmentally sensitive fluorescent dyes. First, Laurdan, a dye which can characterize the phospholipid packing and order in the membrane^74,75^ was placed in the DOTAP: DOPE vesicles which formed the membranous coating on the hydrogel artificial cells (Figure 4H) (Figures S24 and S25). The general polarization of the Laurdan dye was then calculated from the fluorescence emission spectra of the Laurdan dye in vesicles and on the hydrogel artificial cells. A general polarization value of 1 corresponds to a membrane being in a gel state (tightly packed phospholipids) whist a value of −1 corresponds to a membrane being in a fluid state (disordered phospholipids).^74,76^ It was seen that fully coated and partially coated hydrogel artificial cells possessed a membrane which had predominantly fluid characteristics, matching the FRAP results. The general polarization values between the bulk DOTAP:DOPE vesicles (dotted line) and the hydrogel membrane coatings exhibited minor differences, indicating that there may be very small differences in packing in the different environments. Moreover, the general polarization values of the DOTAP:DOPE hydrogel artificial cell membrane were also similar to hydrogels coated with DOTAP:DOPC vesicles and other fluid vesicle controls (DOPC^77^ and DOTAP:DOPC) and different to gel phase 1,2-dihexadecanoyl-*sn*-glycero-3-phosphocholine (DPPC) vesicles,^78^ further indicating membrane fluidity (Table S3). Another dye, BODIPY C_10_ (BC10) which can be used the derive the microviscosity of lipid membranes from its fluorescence lifetime,^79^ was also incorporated in the DOTAP: DOPE vesicles and the membrane coating on the hydrogel artificial cells (Figure 4I) (Figures S26 and S27). Fluorescence lifetime imaging of the membrane dye enabled calculation of the membrane microviscosities.^80^ The microviscosity values of the DOTAP:DOPE membrane coating were similar to a range of other fluid lipid membranes including (DOPC vesicles,^81^ DOTAP:DOPC vesicles and DOTAP:DOPC coated hydrogel artificial cells) and different to analyzed gel phase DPPC vesicles^82^ (Table S4). This again confirmed that the membrane coating was fluid. A slight increase in viscosity was seen when the membrane was attached to the hydrogel artificial cells compared to the DOPE:DOTAP vesicles in bulk (dotted line), this may be due to the underlying hydrogel network reducing the motion of the attached membrane in a similar manner to what was seen for lipid membranes upon cytoskeleton attachment.^83^

Finally, the stability of the hydrogel artificial cell membrane was tested through incubating the hydrogels in fresh sucrose buffer over 2 weeks (Figure S28), in addition to dehydrating and rehydrating a population of hydrogel artificial cells (Figures S29 and S30) (Video S7). Imaging showed that the membrane coating remained attached to the hydrogel over a 2 week period. Furthermore, on dehydration and subsequent rehydration, the membrane coating remained stably attached to the hydrogel and shrunk/swelled in response to the shrinking and swelling of the underlying hydrogel network. The hydrogels could also swell back to almost their original size with the membrane coating attached. By showing that membrane coated hydrogel artificial cells are stable in buffer for an extended period of time and upon drying and rehydrating, we enable the possibly for membrane coated hydrogels to be dried and deployed on rehydration in a wide range of environments in a similar way to other synthetic biology systems.^84^

Overall, through utilizing vesicle fusion events, we can assemble a stable, contiguous and fluid multilamellar lipid membrane of a tailorable penetration depth from a shell a few microns thick to encompassing the entire hydrogel network by varying the vesicle incubation time (Figure 4J). In comparison to previous attempts to produce a lipid membrane around a hydrogel,^39–41^ our membrane is directly incorporated on the entire hydrogel network instead of as a separate component surrounding the hydrogel. Alginate hydrogels have a rough surface on the microscale^85^ and nanoscale.^86^ Thus, this will produce regions of different membrane curvatures, more closely mimicking cell membranes than current systems.^87^ Hence making these hydrogel artificial cells ideal candidates to investigate membrane protein functions through the incorporation of proteoliposomes^88^ into the hydrogel membrane. Moreover, through assembly of the lipid membrane through vesicle fusion, the membrane can be assembled without the inclusion of contaminants such as oils, unlike some other cell membrane models.^89^ Consequently, our membrane provides an excellent building block to develop complex hydrogel based artificial cells and as a model to study membrane functions.

### Enhancement of Hydrogel Artificial Cell Properties through Membrane Addition

After characterizing the hydrogel artificial cell membrane coating, we investigated how the membrane had enhanced the existing properties of the hydrogel structure. As the hydrogel artificial cells are porous to low molecular weight molecules,^58^ initially we investigated how the permeability would be altered upon membrane addition as lipid membranes are impermeable to many small molecules^34^ (Figure 5A–C) (Video S8). We saw that upon application of the membrane coating to cover the entire gel, permeation of small membrane impermeable molecules such as calcein dye^90^ (*M*_W_ ∼ 622 Da) was prevented, thus enabling the hydrogel artificial cells to possess permeability properties similar to other membranous artificial cell models such as giant unilamellar vesicles.^91^ Meanwhile upon the application of a partial membrane coating, calcein could still readily diffuse into the center of hydrogel artificial cells, however exclusion of the small molecule calcein dye could be seen from the region containing the membrane. This effect was more prominent on membrane coatings covering more of the hydrogel network (Figure S31). This shows that in addition to a fully penetrated membrane coating preventing permeation into the hydrogel, a partial membrane coating could be used to exclude small membrane impermeable molecules from regions of the hydrogel. We have therefore created a hydrogel system with different permeabilities to molecules within the same structure. This could be used to achieve two different release rates of a drug, for example through encapsulation within hydrogel artificial cells with differing membrane coating thicknesses, a valuable tool for drug delivery applications.

**Figure 5:**
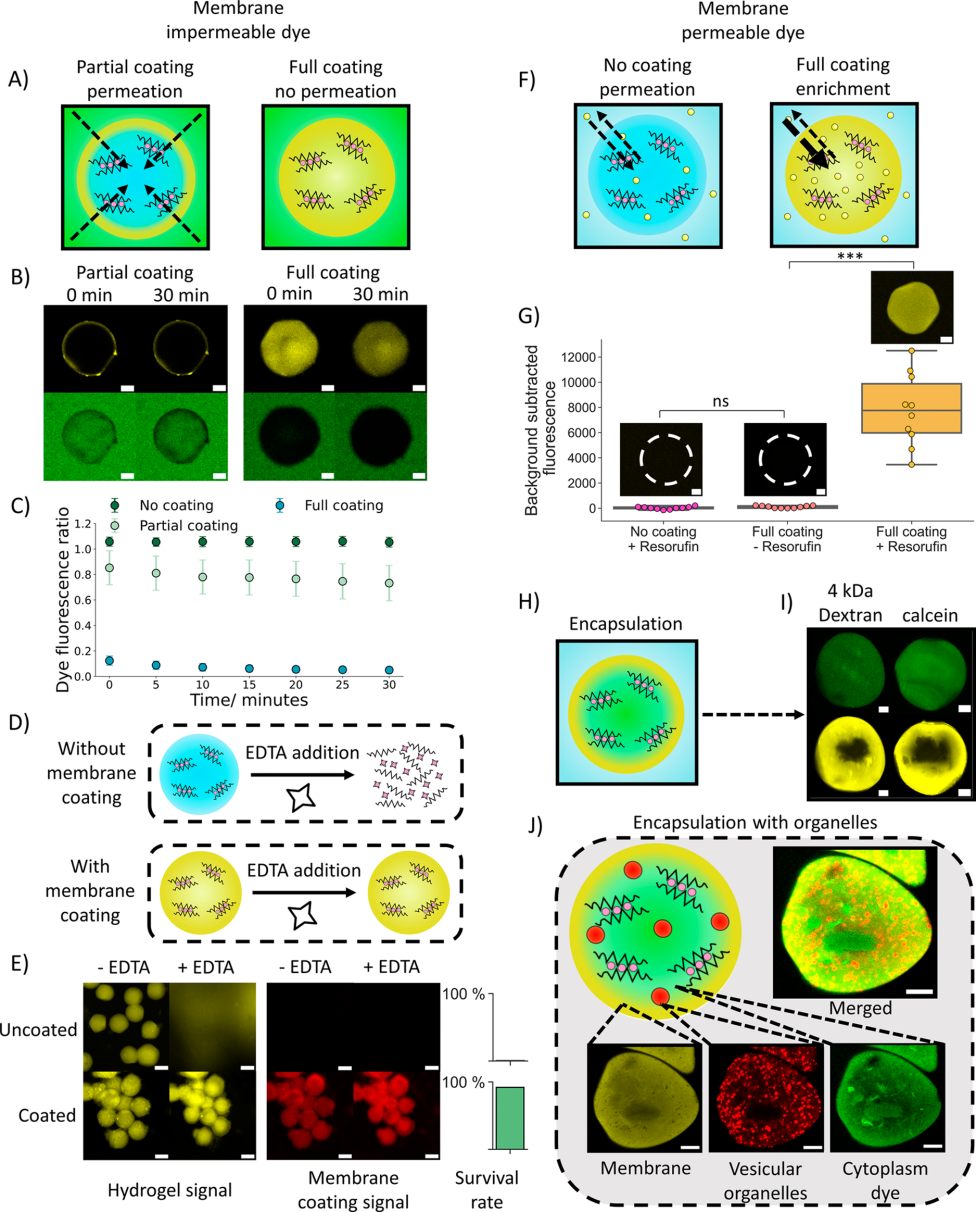
Utilization of the membrane to enhance hydrogel artificial cell properties. (A) Diagrams indicating how permeation of membrane impermeable small molecules can be altered by the thickness of the membrane coating. A partial membrane coating enables permeation while a fully penetrated membrane coating prevents permeation of membrane impermeable small molecules into the hydrogel artificial cells. (B) Confocal microscopy images showing hydrogel artificial cells with a partial and full membrane coating immersed in a calcein containing solution after 0 and 30 min. The full membrane coating prevents calcein permeation into the hydrogel artificial cells. With the partial coating there is dye present in the center of the hydrogel. The membrane coating however does exclude dye in the region it is present, leading to different permeabilities in different portions of the gel. The scale bars are 20 μm. (C) A graph showing how calcein permeates into the hydrogel artificial cells with different sizes of membrane coating. With a full membrane coating there is more dye present externally than internally while with a partial coating the ratio of internal dye to external dye closer to 1, showing increased free permeation into the core of the hydrogel. Over 30 min the dye fluorescence ratios for both coating architectures show limited change. The error bars represent the standard deviation from *n* = 10 hydrogel artificial cells. (D) Illustration depicting the impact of EDTA addition on uncoated and fully coated hydrogel artificial cells. On EDTA addition to uncoated hydrogels, gel dissolution occurs. Meanwhile, the fully coated hydrogels are not dissolved by EDTA due to the protection offered by the membrane coating. (E) Image panels presenting the effects of EDTA addition on uncoated and coated fluorescent hydrogel artificial cells. The yellow fluorescent signal is from the hydrogel while the red fluorescent signal is from the membrane coating. On EDTA addition, the uncoated hydrogels are dissolved, shown by the loss of localized fluorescence, while the coated hydrogel network and membrane coating remain intact. The survival rate graphs depict the percentage of hydrogels present after EDTA addition from a population of *n* = 50. The scale bars on all of the images are 50 μm. (F) Diagrams indicating how permeation of membrane permeable small molecules can be altered by the thickness of the hydrogel membrane coating. (G) Box plots with representative confocal microscopy images showing on addition of a fully penetrated membrane coating, the small molecule Resorufin is enriched in the hydrogel artificial cells compared to the background signal. The dotted circles show the positions of hydrogel artificial cells. The scale bars are all 20 μm and *n* = 10 hydrogel artificial cells were analyzed for each condition. (H) A schematic demonstrating how a penetrated hydrogel artificial cell membrane can be used to encapsulate small molecules in the hydrogel. (I) Confocal microscopy images showing the encapsulation of two different small molecules (calcein and 4 kDa Dextran) in a hydrogel artificial cell (green channel) through the use of a thick membrane coating (yellow channel). The scale bars are 20 μm. (J) An illustration with corresponding confocal microscopy images showing how lipid organelles can be interfaced into the hydrogel artificial cells, creating a structure that has lipid organelles trapped by the hydrogel structure and a separate small molecule dye encapsulated by the membrane in the cytoplasm region. The scale bars are 20 μm.

To highlight the utility of altering the permeability of the hydrogel artificial cells with a membrane coating. We added EDTA (*M*_W_ ∼ 292 Da) to solutions containing fully coated and uncoated hydrogel artificial cells (Figure 5D,E). EDTA can chelate Calcium ions cross-linking the alginate hydrogel structure, leading to dissolution of the hydrogel artificial cells.^92^ On addition of EDTA to uncoated hydrogel artificial cells labeled with fluorescent alginate, we saw destruction of the hydrogel population. However, with the fully coated hydrogel artificial cells, where the hydrogel was labeled with fluorescent alginate and the membrane coating labeled with a membrane based Cy5 tag, 92% of the hydrogel population remained stable upon EDTA addition, confirming that the membrane prevents the permeation of small molecules to the hydrogel network. We attribute the dissolution of a small percentage of this population to possessing incomplete membrane coatings. These findings enable coated hydrogel artificial cells to be used in a range of environments where protection of the hydrogel chassis is required.

To further characterize the permeability of the membrane coating. The small molecule dye Resorufin (*M*_W_ ∼ 213 kDa) which is membrane permeable^93^ and significantly more hydrophobic than the previously analyzed calcein dye^94^ was added to hydrogel artificial cells with a range of differing membrane coating penetration depths. On Resorufin addition we observed that the dye readily permeated uncoated hydrogel artificial cells and permeated into and was trapped in hydrogel artificial cells with a fully penetrated or partially penetrated membrane coating (Figure 5F,G) (Figure S32), unlike the calcein dye which was excluded from the coated hydrogel artificial cells. This further shows that the hydrogel artificial cells possess small molecule permeability heavily influenced by the attached lipid membrane and have permeability characteristics similar to lipid membranes.^95^

We then sought to encapsulate small molecules into the hydrogel artificial cells, a feat that would not be possible without the membrane. After incubation in the vesicle coating solution and dye for 24 h followed by a wash step, it was seen that the small molecule dyes were successfully localized into the hydrogel structure (Figure 5H,I). We further confirmed this entrapment by photobleaching a hydrogel artificial cell with encapsulated material and could see the internal fluorescence of the gel changed in comparison to the exterior solution (Figure S33), confirming separation between the hydrogel artificial cell cargo and the exterior solution. As a result, this creates an environment within the artificial cell where large moieties (e.g., organelles) are held in place by the hydrogel network while smaller moieties are free to diffuse within the artificial cell but are separate to the external environment due to the hydrogel lipid membrane. This platform is similar in organization to the cell cytoplasm and internal organelles found in biological systems.

To demonstrate this cellular similarity, we encapsulated 1-palmitoyl-2-oleoyl-glycero-3-phosphocholine (POPC) lipid vesicles with a membrane based Cy5 tag into the hydrogel artificial cells to act as cellular organelles (choosing this composition to minimize interaction between the organelle membranes and hydrogel matrix). We then demonstrated that the membrane coating could be applied to such a system and not interfere with the stability of the internal vesicular organelles (Figure S34). Again, indicating that the membrane coating is confined to the hydrogel mesh around the hydrogel pores and discrete to internal organelles which sit in the hydrogel pores. This also shows that two lipid membranes with different compositions (the vesicular organelles and coating) can exist within the hydrogel artificial cells, enabling the compositional difference to be exploited to control interactions between the different lipid membranes. To further prove that the membrane coating does not interfere with internal organelles, we placed solid magnetic particles into the hydrogel artificial cells and observed again that the internal magnetic particles remained uncoated and stable on coating application (Figure S35). With this conformation we then encapsulated calcein dye with the membrane coating in the organelle containing system. Upon observation of the produced hydrogel artificial cells (Figure 5J) we could see three separate fluorescent signals, one from the applied membrane, one from the internal vesicular organelles and one from the calcein dye encapsulated in the cytoplasm region. This shows that the hydrogel membrane enhances the properties of the hydrogel structure, can exist orthogonally to other membrane-containing compartments within the same microstructure, and enables the recreation of cell mimics with increasingly accurate organization.

## Conclusion

In conclusion we have produced a methodology for assembling a fluid, stable and biologically relevant lipid membrane coating of a tunable penetration depth and a unique architecture on cell sized hydrogel artificial cells. We then showcased that the assembled membrane could augment the existing properties of the hydrogel by more finely tuning the hydrogel permeability, protecting the hydrogel from degradation by small molecules, and through acting as a scaffold for assembling increasingly complex artificial cell structures. Through these developments we have laid a foundation for the deployment of increasingly biomimetic hydrogel based artificial cells as tools to replicate biological form and function through the incorporation of alternative biological componentry, the inclusion of different phospholipids to improve mimicry of the cell membrane^96^ or through interactions with other cell mimics.^97^ Moreover, future work could aim to utilize the produced lipid membrane with other hydrogel soft matter systems to act as, a biolubricant for alleviation of friction related diseases,^98,99^ a component within bioelectronic devices,^100^ a scaffold for cells to interact with in tissue engineering^101^ and as a mechanism to further improve hydrogel-based drug delivery platforms.^102^

## Materials and Methods

### Materials

The lipids 1,2-dioleoyl-*sn*-glycero-3-phosphoethanolamine (DOPE), 1,2-dioleoyl-3-trimethylammonium-propane (chloride salt) (DOTAP), 1,2-dioleoyl-*sn*-glycero-3-phosphocholine (DOPC), 1,2-dihexadecanoyl-*sn*-glycero-3-phosphocholine (DPPC) and 1-palmitoyl-2-oleoyl-glycero-3-phosphocholine (POPC) were purchased from Avanti Polar Lipids (Alabaster, AL) as powders and used without further purification. The lipids 1,2-dioleoyl-*sn*-glycero-3-phosphoethanolamine-*N*-(Cyanine 5) (Cy5-PE) and 1,2-dioleoyl-*sn*-glycero-3-phosphoethanolamine-*N*-(lissamine rhodamine B sulfonyl) (ammonium salt) (rhodamine-PE) were purchased from Avanti Polar Lipids (Alabaster, AL) and used as chloroform stocks. Polydimethylsiloxane (PDMS) Sylgard 184 elastomer kits were purchased from Dow Corning (Michigan, USA). BODIPY-based molecular rotor BC10 was previously synthesized according to published procedures.^103^ 6-Dodecanoyl-2-dimethylaminonaphthalene (Laurdan dye) and the membrane dyes 1,1′-dioctadecyl-3,3,3′,3′-tetramethylindocarbocyanine perchlorate (Dil) and DiOC_18_(3) (3,3′-dioctadecyloxacarbocyanine perchlorate) (DiO) were purchased from Thermo Fisher Scientific (UK). Stock solutions of Laurdan and BC10 were prepared at 3 mM in chloroform. Rhodamine B-labeled alginate was purchased from HAworks (New Jersey, USA). The MagneHis Ni-Particles were obtained from Promega (Southampton, UK). All other reagents including sodium alginate, chloroform, sucrose, HEPES buffer, potassium chloride, calcium chloride, ethylenediaminetetraacetic acid (EDTA), sorbitan monooleate (Span 80), mineral oil, calcein dye, fluorescein isothiocyanate–dextran (4 kDa), resorufin and acetic acid were obtained from Sigma-Aldrich (Gillingham, UK) unless specified.

### Production of Lipid Vesicles

Lipid films were initially made by weighing out appropriate amounts of lipid and dissolving them in chloroform. In all lipid compositions (1:1 molar ratio of DOTAP:DOPE, 1:1 molar ratio of DOPE:DOPC, 1:1 molar ratio of DOTAP:DOPC, DOPC, DPPC and POPC) the total amount of lipid used was 5 mg. For experiments containing a fluorescent lipid or dye label (Cy5, Rhodamine, DiO or Dil) these dyes were added at 0.3 mol %, the DiO and Dil dyes were only used in experiments S11, S15 and S18. Meanwhile the fluorescent probes (Laurdan and BODIPY) were added at 0.5 mol % to the lipid composition. The lipid in chloroform solutions were mixed for 1 min to ensure lipid mixing before the chloroform was gently evaporated under a stream of N_2_. The films were then left under vacuum overnight to remove the residual chloroform.

The lipid films were then rehydrated with 0.5 M sucrose, 100 mM KCl, 100 mM HEPES (pH 7.4) and 20 mM CaCl_2_. For the dextran encapsulation experiment (Figure S15) this solution had 1 mM 4 kDa fluorescein isothiocyanate–dextran added to it. Meanwhile for the lipid films included as Cy5 labeled organelles, the lipid film was rehydrated with just 0.5 M Sucrose and 100 mM KCl. The final concentration of all the lipid solutions was 10 mg mL^–1^.

The lipid solutions were then freeze–thawed 5 times by flash freezing the vesicle suspension in liquid N_2_, thawing the sample by heating to 50 °C and finally vortexing for 30 s. The vesicles were then extruded through 0.1 μm polycarbonate membranes 21 times to produce a vesicle population of ∼120 nm in size (Figure S3) for use either for encapsulating as organelles within the hydrogels or for incubating with the hydrogel to produce the lipid membrane.

### Manufacture of Microfluidic Devices

The PDMS devices were prepared using standard soft lithography techniques with device designs adapted from Trantidou et al.^104^ The silicon master wafers (Inseto) were first created by depositing a photoresist (SU-8 3050, Kayaku Advanced Materials, MA, USA) with a spin coater. The wafers were then baked before UV light exposure (365 nm, 300 mJ cm^–2^) through an acetate photomask (Micro Lithography services, UK) containing the device design. After a post exposure bake, the unexposed features were removed using propylene glycol monomethyl ether acetate developer and rinsed with Isopropyl alcohol. Upon production of the final pattern, the wafers were silanized with trichloro­(1*H*,1*H*,2*H*,2H-perfluorooctyl)­silane under vacuum overnight.

The patterned wafers then had degassed PDMS (10:1 elastomer/curing agent) poured onto them and were left to cure for at least 3 h at 60 °C. The patterned PDMS was then removed from the underlying wafer, 1.5 mm holes were punched for the inlet and outlet ports before being irreversibly bonded to a glass slide in order to seal the microfluidic channels. This was accomplished by exposing both the glass slide and patterned side of the PDMS to plasma (Harrick Plasma, NY, USA) for 1 min before contacting the surfaces together. The devices were then left overnight before use to ensure complete bonding between the glass slide and PDMS.

### Microfluidic Production of Hydrogels

The 2 wt % microscale hydrogels were produced by first making stock solutions of 2 wt % alginate, 0.5 M sucrose and 50 mM Ca-EDTA. For the experiment using 0.5 wt % alginate hydrogels, a solution of 0.5 wt % alginate, 0.5 M sucrose and 50 mM Ca-EDTA was used to prepare the hydrogels. For the inclusion of magnetic particle organelles, magnetic particles diluted 100-fold from the purchased stock were added to the 2 wt % alginate stock solution. When the POPC Cy5 vesicle organelles were included in the hydrogel interior, a solution of 4 wt % alginate, 100 mM Ca-EDTA and 0.5 M Sucrose was diluted by the produced Cy5 vesicles to yield a solution containing 2 wt % alginate, 0.5 M sucrose, 50 mM Ca-EDTA, 50 mM KCl and the Cy5 vesicle organelles. When a fluorescent signal was required from the hydrogel structure, fluorescently labeled alginate was added at a concentration of 0.1 wt % to the stock solution.

The oil phases for the microfluidics had the compositions of 5 wt % Span 80 in mineral oil and 1 V/V % acetic acid and 5 wt % Span 80 in mineral oil. The addition of Span 80 into the oil phases increased the stability of the aqueous droplets and thus limited coalescence and formation of larger than intended alginate hydrogels. The acetic acid was used in the second oil stream to gelate the aqueous droplets into alginate hydrogels in this region.

The aqueous and oil phases were then added into the appropriate inlets of the PDMS microfluidic device along with the outlet tubing (Figure S1). Polyethylene tubing (Kinesis, UK) was used to deliver solutions both to and from the microfluidic chip and a pressure pump (Elveflow, Paris, France or Fluigent, Paris, France) was utilized to control the flow rates of the aqueous and oil phases. The first oil phase flow rate was always larger than that of the aqueous flow rate to ensure droplet formation occurred. The size of the aqueous droplets was dictated by the channel geometries and the flow rate ratio of the aqueous to first oil phase thus enabling a range of different size gels to be produced. The produced hydrogels were collected by outlet tubing from the microfluidic device connecting to an Eppendorf tube.

The collected hydrogels were then centrifuged for 5 min at 9000*g* to produce a pellet with an oil phase supernatant that was removed. The pellet could then be stored in the fridge. Upon use the pellet was resuspended in sucrose buffer (0.5 M sucrose, 100 mM HEPES, 100 mM KCl, 20 mM CaCl_2_ pH 7.4) where the additional calcium prevented any unwanted hydrogel degradation from occurring. This buffer had an osmolarity of ∼860 mOsmol kg^–1^. The sucrose contained in all the solutions was added to enable the hydrogel artificial cells to contain lipid organelles with encapsulated dyes and to allow compatibility with other artificial cell structures, i.e. giant unilamellar vesicles.^105^

### Microfluidic Microscopy

A NikonTE2000-U microscope with a Ximea MQ013MG-E2 camera and a 4× objective was used to record the brightfield timelapse videos and images for the microfluidic production of hydrogels.

### Membrane Assembly on Hydrogel Artificial Cells

To assemble membranes on the hydrogel artificial cells and larger hydrogels, the bare hydrogels were incubated with the desired lipid vesicle composition (typically DOTAP:DOPE with 0.3 mol % rhodamine-PE) at a concentration of 5 mg mL^–1^. If the membranous hydrogels required to be resuspended after being coated, they were centrifuged for 5 min at 9000*g*. The supernatant was again discarded and the hydrogels with a membrane coating were resuspended in fresh buffer (0.5 M Sucrose, 100 mM KCl, 100 mM HEPES, 20 mM CaCl_2_) where no lipids were present.

### Dynamic Light Scattering and Zeta Potentials of Lipid Vesicles and Hydrogels

A Malvern Zetasizer Ultra instrument (Malvern, UK) with a 632.8 nm HeNe gas monochromatic laser was used to size the vesicles that formed the hydrogel membrane through using back scattered light. Scattered light was detected at an angle of 173° from the transmitted beam to minimize unwanted reflection. The vesicle samples were diluted in a 1:10 ratio in the sucrose buffer (0.5 M sucrose, 100 mM HEPES, 100 mM KCl, 20 mM CaCl_2_ pH 7.4).

For zeta potential measurements, samples were diluted in a 1:10 ratio in the sucrose buffer (0.5 M sucrose, 100 mM HEPES, 100 mM KCl, 20 mM CaCl_2_ pH 7.4) and placed in folded capillary zeta cells (Malvern). The analysis mode selected was monomodal. The coated hydrogels analyzed through this procedure had been washed and resuspended in buffer to minimize vesicles in the coating solution from interfering with the measurements.

### Confocal Microscopy of Hydrogel Coatings

A Leica stellaris 8 was used for all confocal imaging unless specified. 10× and 20× objectives were used to image the various fluorophores with a WLL (white light) laser and emissions recorded through HyD detectors. The pinhole was set to a diameter of 1 Airy unit. The lasers were set to excite the fluorophores at their excitation maxima and the detectors to record emission at their emission maxima. For the fluorophores used with this confocal microscope these values were, 490/520 nm (fluorescein isothiocyanate–dextran), 495/514 nm (calcein), 560/583 nm (rhodamine-PE), 571/584 nm (Resorufin) and 639/663 (Cy5-PE). The 8 h timelapses were taken using a Zeiss LSM700 and a 40× objective with a pinhole set to 1.33 Airy units. Again, the lasers were set to excite the fluorophores at their excitation maxima and the detectors to record emission at their emission maxima. For the fluorophores used with this confocal microscope these values were 560/583 nm (Rhodamine-PE). Samples were imaged by placing the hydrogels into PDMS imaging chambers sandwiched between coverslips.

Experiments S11, S15 and S18 were performed using an Olympus IXplore IX85 Spin microscope. Again, 10× and 20× objectives were used to image fluorophores excited by 488 nm (for DiO) and 561 nm (for Dil) lasers with the emission recorded after passing through either 525/50 nm or 617/73 nm filter cubes respectively using an ORCA-Quest 2 qCMOS camera. Samples were imaged by placing the hydrogels into either μ-slide 18-well glass bottom chambers (ibidi) or a transparent 96 well plate.

All Images were analyzed using FIJI. All statistical analysis was performed using the SciPy.stats library in python using an unpaired *t*-test with unequal sample variances. The p values were set to the following levels: *< 0.05, ** < 0.01 and *** < 0.001 with >0.05 being not significant (ns).

The membrane to background fluorescence ratio was calculated through the following equation by taking a region of the membrane (*F*_membrane_) and dividing it by a local background region (*F*_background_).

1 $$Membrane\;to\;background\;fluorescence\;ratio=\frac{F_{membrane}}{F_{background}}$$

The line profile data was normalized using the below equation where *F*_max_ is the maximum fluorescence signal and *F*_*x*_ is the fluorescence at a given point on the line taken.

2 $$Normalised\;fluorescence/intensity=\frac{F_{x}}{F_{\max}}$$

The average membrane intensity was calculated by averaging the intensity of membranous region on the hydrogels.

The normalized hydrogel area was calculated using the following equation. *A*_0_ was the area at the start of the experiment and *A*_*t*_ was the area at a given time point.

3 $$Normalised\;hydrogel\;area=\frac{A_{t}}{A_{0}}$$

The normalized fluorescence of the hydrogels was obtained through eq 4 where *F*_max_ is the maximum fluorescence signal before the bleaching and *F*_*t*_ is the fluorescence at a given time point.

4 $$Normalised\;fluorescence=\frac{F_{t}}{F_{\max}}$$

The percentage of hydrogel area coated was calculated through measuring the area of the entire hydrogel (*A*_hydrogel_) and dividing by the area of the hydrogel occupied by the membrane coating (*A*_membrane_). This area was determined by thresholding the coated hydrogel artificial cells.

5 $$Hydrogel\;area\;coated\;\left(\%\right)=\frac{A_{membrane}}{A_{hydrogel}}\times 100$$

The membrane coverage change was calculated through measuring the area of the entire hydrogel before and after a period of time (*A*_*t* hydrogel_ and *A*_0 hydrogel_) and dividing by the area of the hydrogel occupied by the membrane coating before and after the same period of time (*A*_*t* membrane_ and *A*_0 membrane_). The areas were determined by thresholding the coated hydrogel artificial cells.

6 $$Membrane\;coverage\;change\;\left(\%\right)=\left(\frac{A_{t\;membrane}}{A_{t\;hydrogel}}-\frac{A_{0\;membrane}}{A_{0\;hydrogel}}\right)\times 100$$

The lipid concentration on the hydrogel artificial cells was calculated by fitting a linear fit to a lipid concentration calibration curve. The fluorescence intensity of the hydrogel artificial cells was then converted to lipid concentration using this fit (Figure S14).

For the fluorescence recovery after photobleaching experiments, the fluorophores were photobleached using 100% laser power for the bleaching time. The photobleaching data was then normalized using eq 4.

### Widefield Microscopy

A Nikon eclipse Ti_2_–U inverted microscope with a CoolLED pE-300^white^, a 4×, 10× or 20× objective with a 1.5× magnification applied and a Nikon DS-Qi2 camera was used for widefield imaging. The fluorescence images were collected with a TRITC-B or a Cy5-4040C filter cube. Images collected with this widefield microscope were Figure 5E, S13, S21, S29, S30 and S35 and Video S7.

### Cell Cytotoxicity Assay

HEK 293 cells were obtained from the American Type Culture Collection (ATCC) and maintained in DMEM (Dulbecco’s Modified Eagle Medium, PAN Biotech, 04-03591) supplemented with 10% fetal bovine serum (FBS; PAN Biotech, P30-3031), 1% penicillin–streptomycin (10,000 U/mL; Gibco, 15140122), and 12.5 mM HEPES. Cells were cultured at 37 °C in a humidified incubator with 5% CO_2_ (PHCbi, MCO-170AICUVD-PE).

The cytotoxicity of the hydrogel artificial cells toward HEK 293 cells was evaluated using a MTT assay. Briefly, cells were seeded at a density of 2 × 10^5^ cells/mL in 100 μL medium per well in a 96-well plate and incubated overnight at 37 °C and 5% CO_2_. After incubation, the culture medium was replaced with 100 μL of MTT solution (0.5 mg/mL), followed by a 4 h incubation under the same conditions. The MTT-containing medium was then carefully removed, and 100 μL of DMSO was added to each well to dissolve the formazan crystals. The plate was placed on an orbital shaker for 15 min, and absorbance was measured at 570 nm using a plate reader (Spark, Tecan Life Science). Wells containing medium alone without cells served as blanks (*A*_blank_). The equation below was used to calculate the viability of the cells

7 $$Viability\;\left(\%\right)=\left(\frac{A_{cells+hydrogels}-A_{blank}}{A_{cells}-A_{blank}}\right)\times 100$$

### Production of Millimeter Sized Hydrogels

Millimeter sized hydrogels were produced through adding a 10 V/V % solution of acetic acid to the 2 wt % alginate solution (2 wt % alginate, 50 mM Ca-EDTA, 0.5 M sucrose) in a 1:10 ratio (to make a final concentration of 1 V/V % acetic acid). The gels were then washed with buffer (0.5 M sucrose, 100 mM KCl, 100 mM HEPES, 20 mM CaCl_2_) to remove excess acetic acid.

### Small and Wide-Angle X-ray Scattering

The millimeter hydrogel samples after being coated with a membranous coating for either 2 or 24 h were resuspended and immersed in fresh buffer containing no lipids (0.5 M sucrose, 100 mM KCl, 100 mM HEPES, 20 mM CaCl_2_) and placed into 1.8 mm inner diameter polycarbonate capillaries that were sealed with epoxy resin. Small and wide-angle X-ray diffraction data was then obtained from beamline I22 at Diamond Light Source. Diffraction patterns were collected using an X-ray energy of 18 keV (wavelength 0.689 Å) and a sample to detector distance of 213 mm for the WAXS and 7727 mm for the SAXS. The raw data was then processed using a pipeline from the beamline technicians at Diamond.^106^

### Atomic Force Microscopy on Hydrogel Artificial Cells

Hydrogel artificial cells were dried on a Teflon surface before being partially embedded in two-part epoxy by thinly spreading a freshly mixed 5 min epoxy (DevCon) on a metal stub, leaving it to minimally cure for 2 min, then pressing lightly against the Teflon and hydrogel surface. After the epoxy layer was fully cured it was separated from the Teflon, leaving hydrogels part embedded in the epoxy. The sample was then hydrated with buffer containing no lipids (0.5 M sucrose, 100 mM KCl, 100 mM HEPES, 20 mM CaCl_2_) prior to characterization and inserted into the Multimode 8 AFM with Nanoscope V controller (Bruker, Santa Barbara, CA). A single MLCT-BIO-C cantilever C (nominal tip radius 20 nm, nominal spring constant 0.01 N/m) was used for all measurements, calibrated using the thermal noise method in air (*k* = 0.011 N/m). Deflection sensitivity was calibrated against the mica (for DOPE:DOTAP on mica) or clean epoxy around an embedded hydrogel particle.

To apply the lipid coating before insertion into the AFM fluid cell, a solution containing 10 mg mL^–1^ of DOTAP:DOPE vesicles in buffer (0.5 M sucrose, 100 mM KCl, 100 mM HEPES, 20 mM CaCl_2_) was added to the hydrogel artificial cells embedded in epoxy or blank mica for 15 min before several wash cycles were performed with fresh buffer without lipids to remove excess unbound vesicles. Force curves were acquired at 1.0 mm s^–1^ velocity, and a trigger value between 0.5 and 1.5 nN (50–150 nm deflection) adjusted to ensure tip penetration through to substrate. Force curves were processed in Nanoscope Analysis v3.0 (Bruker), with baseline correction applied. Force curves are presented as force vs tip–sample distance curves, with the contact point set to zero, the contact point found by extrapolation of the linearized Sneddon contact mechanics model. Elastic moduli were determined by fitting with the Sneddon model, using a 20 nm tip radii and the MLCT probe geometry.

### General Polarization Microscopy

Spectral scans of the hydrogel artificial cells with various coating thicknesses (At 24 and 0 h membrane coating) and vesicles containing Laurdan dye were obtained using a Leica TCS SP5 II inverted confocal microscope with the pinhole set to 600 μm on a 20× dry objective (NA: 0.7). Two-photon excitation was provided by a Ti:sapphire laser (Chameleon Vision II, 80 MHz, Coherent) at 760 nm and emission was collected between 395 and 700 nm using a single-photon counting module (SPC830, Becker & Hickl GmbH) across 512 × 512 pixels. All samples were measured at room temperature, below the gel–fluid transition of DPPC vesicles.^107^

The general polarization (GP) of the Laurdan dye in each environment was then calculated using the following eq (eq 8) where *I*_440_ was the membrane fluorescence intensity at 440 nm and *I*_490_ was the intensity at 490 nm.

8 $$GP=\frac{I_{440}-I_{490}}{I_{440}+I_{490}}$$

The normalized intensity values were obtained using the below equation where *I*_max_ is the maximum intensity, *I*_λ_ is the intensity at a given wavelength and *I*_min_ is the minimum intensity.

9 $$Normalised\;intensity=\frac{I_{\lambda }-I_{\min}}{I_{\max}-I_{\min}}$$

### Fluorescence Lifetime Imaging Microscopy

Fluorescence lifetime micrographs of the hydrogel artificial cells with various coating thicknesses. 24 and 0 h membrane coated hydrogels and vesicles containing the BODIPY dye were obtained using the same microscope as for the general polarization microscopy. Two-photon excitation was provided by a Ti:sapphire laser (Chameleon Vision II, 80 MHz, Coherent) at 930 nm and emission was collected between 500 and 580 nm using a single-photon counting module (SPC830, Becker & Hickl GmbH) across 256 × 256 pixels with 256 channels. An instrument response function (IRF) was measured via second harmonic generation from urea crystals. All samples were measured at room temperature, below the gel–fluid transition of DPPC vesicles.^107^

Lifetimes were calculated by fitting the pixel-wise time-resolved lifetime decays to a monoexponential or biexponential (gel-phase DPPC only) model within SPCImage (Becker & Hickl GmbH), resulting in fits with χ^2^ < 1.5. The longer lifetime component was used as a descriptor of viscosities for decays fitted with a biexponential model.^108^

Viscosity (η) was calculated using the following equation^80^ (eq 10) with a known lifetime (τ) value.

10 $$\log_{10}\eta =\frac{\log_{10}\tau +0.75614}{0.4569}$$

The normalized count values were obtained using the below equation where *c*_max_ is the maximum count value and *c*_*t*_ is the number of counts at a given time.

11 $$Normalised\;counts=\frac{c_{t}}{c_{\max}}$$

### Hydrogel Artificial Cell Dehydration and Rehydration

Hydrogel artificial cells were coated with a DOTAP:DOPE lipid membrane for 3 h before being placed in an oven for 2 h at 60 °C to dehydrate the hydrogels. The dehydrated hydrogels were then imaged before being rehydrated with buffer without lipids (0.5 M sucrose, 100 mM HEPES, 100 mM KCl, 20 mM CaCl_2_ pH 7.4) where further imaging was conducted. The normalized diameter of the hydrogels was calculated with the following equation. *d*_initial_ corresponded to the diameter of the hydrogels before dehydration while *d*_dehydrated/rehydrated_ corresponded to the diameter of the hydrogels after dehydration or subsequent rehydration.

12 $$Normalised\;diameter=\frac{d_{dehydrated/rehydrated}}{d_{initial}}$$

### Hydrogel Artificial Cell Membrane Permeation and Encapsulation

To investigate permeation the fully coated hydrogel artificial cells (incubated with DOTAP:DOPE vesicles containing 0.3 mol % Rhodamine-PE for 24 h) and the partially coated hydrogels (incubated for 1 h) were resuspended in fresh buffer containing no lipids (0.5 M Sucrose, 100 mM KCl, 100 mM HEPES, 20 mM CaCl_2_) with 0.25 mM calcein dye. For the Rhodamine permeation experiments hydrogel artificial cells with a full DOTAP: DOPE coating or partial (incubated for 1 h) DOTAP:DOPE coating were resuspended in fresh buffer containing no lipids (0.5 M sucrose, 100 mM KCl, 100 mM HEPES, 20 mM CaCl_2_) with 0.25 mM Resorufin. There was no fluorescent label in the membranous coating. The background subtracted fluorescence for the hydrogels was then calculated using the following equation

13 $$Background\;subtracted\;fluorescence=F_{hydrogel}-F_{background}$$

The calcein dye fluorescence ratio for the permeability experiments were calculated using the following equation where Calcein_Internal_ is the fluorescence intensity in the center of the hydrogel and Calcein_External_ is the fluorescence intensity in the surrounding solution.

14 $$Dye\;fluorescence\;ratio=\frac{{Calcein}_{Internal}}{{Calcein}_{External}}$$

The EDTA degradation experiments were performed by either adding 100 mM of EDTA to fluorescently labeled hydrogels or fluorescently labeled hydrogels that had undergone 24 h of coating with DOTAP:DOPE vesicles containing a fluorescent Cy5 lipid.

The survival rate was calculated by analyzing a population of hydrogels before and after EDTA addition and counting the number of the same hydrogels remaining after EDTA addition.

To encapsulate small molecules within the membrane coating, 0.25 mM calcein or 1 mM 4 kDa fluorescein isothiocyanate-dextran were added to the membrane coating solution and incubated for 24 h before being resuspended in fresh buffer containing no lipids (0.5 M sucrose, 100 mM KCl, 100 mM HEPES, 20 mM CaCl_2_) and imaged. The same procedure was used to encapsulate the calcein dye in the hydrogel artificial cells containing Cy5 lipid vesicle organelles.

## Supplementary Material



















## Notes

Additional data outlining the microfluidic production process of the hydrogels, additional images and control experiments on membrane coated hydrogels, additional FRAP experiments on membrane coatings of different penetration depths and compositions, AFM control experiments, images and tables supporting the Laurdan and molecular rotor experiments, cell viability studies with membranous hydrogel artificial cells, extra dehydration/rehydration and stability experiments performed on the membrane coated hydrogels and further characterization of the membrane coating impacting small molecule permeability and organelle interfacing (PDF)
Video S1: Microfluidic production of hydrogels. The scale bar is 200 μm (AVI)
Video S2: 8 h fluorescence timelapse of DOPE:DOTAP SUV incubation with a population of hydrogel artificial cells. The scale bar is 50 μm­(AVI)
Video S3: 8 h fluorescence timelapse of DOPE:DOTAP SUV incubation with a single hydrogel artificial cell. The scale bar is 20 μm (AVI)
Video S4: Recovery of a DOTAP:DOPE membrane on a hydrogel artificial cell. The scale bar is 20 μm (AVI)
Video S5: Recovery of a DOTAP:DOPC membrane on a hydrogel artificial cell. The scale bar is 20 μm (AVI)
Video S6: Recovery of a partially penetrated DOTAP:DOPE membrane on a hydrogel artificial cell. The scale bar is 20 μm (AVI)
Video S7: Rehydration of membrane coated hydrogel artificial cells. The scale bar is 100 μm (AVI)
Video S8: Prevention of calcein permeation into fully coated hydrogel artificial cells. The scale bar is 20 μm (AVI)
