# Supplementary material for: Assembling Lipid Membrane Scaffolds on Microgel-Based Artificial Cells through Vesicle Fusion onto the Hydrogel Network
Source: ACS Nano. 2026 Jan 8;20(2):1945–61. doi: 10.1021/acsnano.5c12532 (PMC12825382; doi:10.1021/acsnano.5c12532)
Supplement: Supplementary file 1 [file nn5c12532_si_001.pdf]

Supplementary information

Assembling lipid membrane scaffolds on microgel-based artificial cells through vesicle fusion onto the hydrogel network

Author list

Matthew E. Allen[1,2,3,4,5], James W. Hindley[1,2,4], Maya I. Müller[1,2], Kexin Cai[5], Marina K. Kuimova[1,2], Robert V. Law[1,2,4], Simon D. Connell[6], Seraphine V. Wegner[5], Oscar Ces[1,2,4], Yuval Elani[2,3,4]*

[1]Department of Chemistry, Imperial College London, Molecular Sciences Research Hub, 82 Wood Lane, London, W12 0BZ, UK
[2]Institute of Chemical Biology, Imperial College London, Molecular Sciences Research Hub, 82 Wood Lane, London, W12 0BZ, UK
[3]Department of Chemical Engineering, Exhibition Road, Imperial College London, South Kensington, London SW7 2AZ, UK
[4]FabriCELL, Imperial College London, Molecular Sciences Research Hub, 82 Wood Lane, London, W12 0BZ, UK
[5]Institute of Physiological Chemistry and Pathobiochemistry, University of Münster, Waldeyerstraße 15, 48149 Münster, Germany
[6]School of Physics and Astronomy, University of Leeds, Leeds, LS2 9JT, UK

Supporting notes, figures and tables

Supporting notes

Supporting note 1- analysing the structure of the membranous coating

The small angle X-ray scattering spectra (SAXS) obtained in figure 4C contains no additional scattering peaks, therefore making identification of the structure of the membranous coating through the SAXS spectra difficult. However, through identifying a multilamellar structure using the atomic force microscopy (AFM) (**Fig. 4F**), the SAXS spectra can be assigned a lamellar phase. Converting the d spacing value of the scattering peak yields a d spacing of 5.4 nm, which is similar to other membranous structures containing DOPE and DOTAP[1]. Furthermore, this matches the sizes of the bilayer breakthrough events present in the AFM traces (**Fig. 4F and Fig. S23**), confirming that the multilamellar coating is comprised of lipid bilayers of a similar thickness to a cell membrane (3-4 nm)[2].

Supporting tables

Manuscript	Hydrogel size	Membrane architecture	Penetration depth	Stability	Fabrication complexity
This study	Microscale	Multilamellar	Can be varied	At least 2 weeks	Simple- small unilamellar vesicle fusion onto alginate hydrogels
Li et al[3]	Microscale	Lipid bilayer	No penetration	At least 10 minutes	Difficult- Double emulsion microfluidics followed by

					photopolymerisation
Walther et al[4]	Microscale	Lipid bilayer	No penetration	Tens of minutes	Medium- requires a confocal microscope to individually polymerise each giant unilamellar vesicle
Saleem et al[5]	Microscale	Multilamellar	No penetration	Not measured	Medium- small unilamellar vesicle fusion onto a core shell microgel made through custom synthesis
Saeki et al[6]	Microscale	Adhered lipids	No penetration	Not measured	Simple- inverted emulsion transfer of hydrogel particle
Llopis-Lorente et al[7]	Microscale	Lipid bilayer	No penetration	Not measured	Medium- inverted emulsion transfer followed by a pH triggered polymerisation
Hettiarachchi et al[8]	Microscale	Not characterised	No penetration	Months	Difficult- Double emulsion microfluidics followed by hydrogel curing
Wang et al[9]	Microscale	Microgels adhered to a lipid bilayer/ Adhered lipid around a microgel	No penetration	Not measured	Simple- Addition of giant and small unilamellar vesicles to PNIPAM microgels
Tanaka et al[10]	Microscale	Continuous lipid membrane (no details on lamellarity)	No penetration	Not measured	Medium- Addition of small unilamellar vesicles to a fabricated hydrogel microarray
Chin et al[11]	Microscale	Continuous supported lipid membrane (no details on lamellarity)	No penetration	Not measured	Simple- small unilamellar vesicle adhesion to a hydrogel
Rahni et al[12]	Microscale	Not characterised	No penetration	Not measured	Simple/medium- small multilamellar vesicle adhesion to synthesised hydrogels/ photopolymerisa

					tion inside giant vesicles
Versluis et al[13]	Macroscale	Lipid bilayer vesicles surrounded by a hydrogel	Embedded vesicles	Not measured	Simple- Hydrogel formation around lipid vesicles
Tam et al[14]	Macroscale	Lipid bilayer vesicles surrounded by a hydrogel	Throughout the entire hydrogel as discrete vesicles	Not measured	Simple- Lipid vesicles were pipetted onto a hydrogel and left to diffuse in

Table S1: Comparison of processes to produce a membranous coating around hydrogel artificial cells. This study uniquely offers a customisable penetration depth for the membranous coating with a straightforward assembly strategy.

Material	Zeta potential/ mV
Uncoated hydrogels	-11.13
Coated hydrogels	+15.99
Buffer solution	-1.41
DOTAP: DOPE vesicles	+16.31

Table S2: Zeta potentials of materials and buffers used in coated hydrogel artificial cell construction. The uncoated hydrogels and DOTAP: DOPE vesicles are opposite charges and so will be electrostatically attracted to each other. Upon formation of a membrane coating the zeta potential of the hydrogels changes to match that of the DOTAP: DOPE vesicles that comprise the coating, indicating that a coating comprising of this vesicle composition is on the surface of the hydrogel artificial cells.

Material	Laurdan General Polarisation value
DOPC vesicles	- 0.134 ± 0.015
DPPC vesicles	+ 0.155 ± 0.002
DOTAP: DOPC vesicles	- 0.115 ± 0.003
DOTAP: DOPE vesicles	- 0.231 ± 0.006
DOTAP: DOPC coated hydrogels	- 0.267 ± 0.028
DOTAP: DOPE partially coated hydrogels	- 0.186 ± 0.0163
DOTAP: DOPE fully coated hydrogels	- 0.250 ± 0.021

Table S3: Laurdan General Polarisation value comparison. All hydrogel samples and vesicle controls measured in this study apart from DPPC had a negative value, indicating that the DOTAP: DOPE hydrogel membranes possessed fluid characteristics. The errors correspond to the standard deviation of n=3 regions analysed for the bulk vesicles and n=15 hydrogels for the hydrogel artificial cell samples.

Material	Viscosity/ cP
DOPC vesicles	124.8 ± 1.1
DPPC vesicles	258.8 ± 10.3
DOTAP: DOPC vesicles	121.8 ± 3.0
DOTAP: DOPE vesicles	138.3 ± 7.9
DOTAP: DOPC coated hydrogels	119.04 ± 1.3
DOTAP: DOPE partially coated hydrogels	153.9 ± 2.5
DOTAP: DOPE fully coated hydrogels	154.2 ± 2.4

Table S4: Viscosity value comparison. All hydrogel samples and vesicle controls measured in this study apart from DPPC had a similar viscosity value, indicating that the DOTAP: DOPE hydrogel membranes possessed fluid characteristics. The errors correspond to the standard deviation of n=3 regions analysed for the bulk vesicles and n=15 hydrogels for the hydrogel artificial cell samples.

Supporting figures

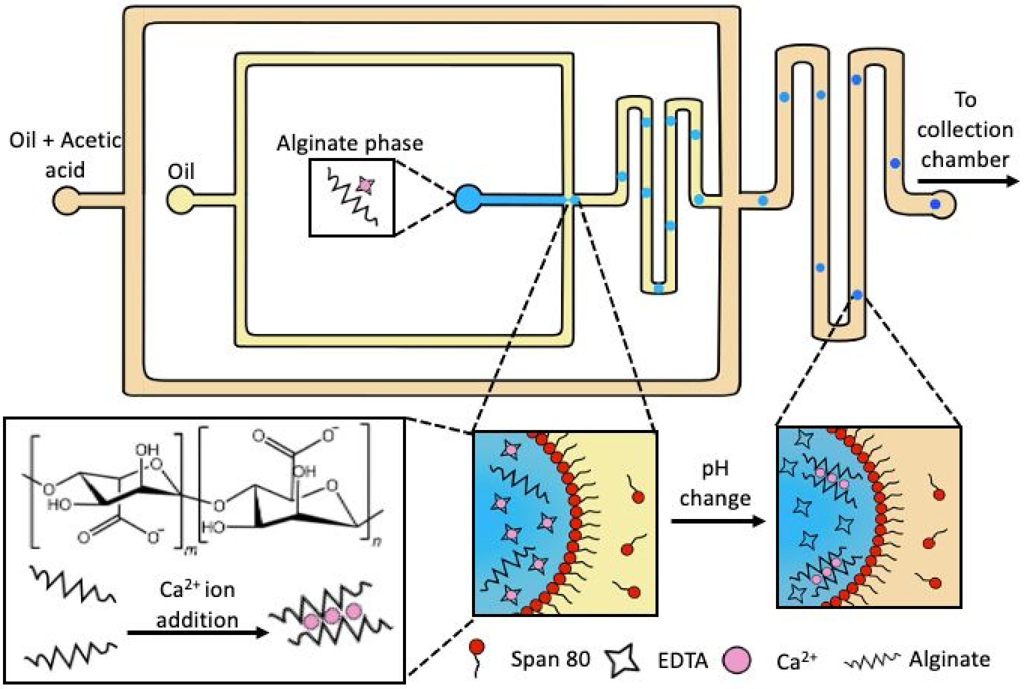

Figure S1: Schematic of the microfluidic process used to make the alginate hydrogels. The device has 3 inlets; one aqueous phase where the alginate precursor solution is added (with/ without vesicular organelles) and two oil phases, one for mineral oil + 5wt% Span 80 and one for mineral oil + 5wt% Span 80 + 1V/V% Acetic acid. The aqueous phase will meet the first oil phase where hydrogel precursor droplets shall be produced. These droplets will then meet a second oil phase where the acetic acid will drop the pH of the droplets, thus enabling the calcium ions to dissociate from EDTA and bind to the alginate, forming the hydrogels. The hydrogels are then collected at the end of the device.

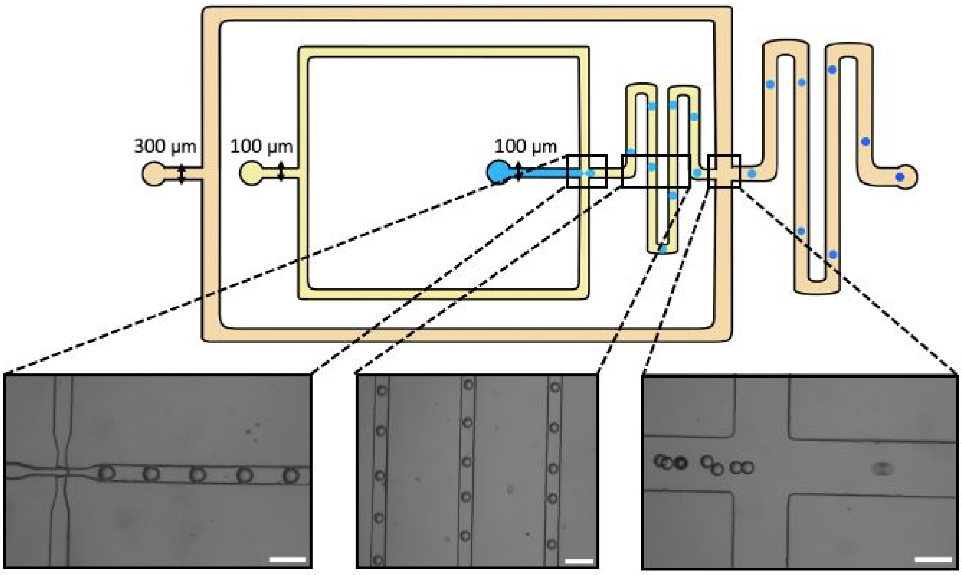

Figure S2: Production of hydrogel artificial cells. A schematic with associated microscopy images showing the creation of the artificial cells. Stabilised hydrogel precursor droplets are first formed before being carried to a second oil phase where the hydrogels are gelated. Through altering the flow rates a range of different sizes can be created. The microfluidic devices used were adapted from *Trantidou et al*[15] where the depth of the channels was 100 µm at the first aqueous/oil flow focussing junction and 200 µm at the second oil/ oil junction. The dimensions of the three main channels are shown on the schematic. The scale bars are 200 µm.

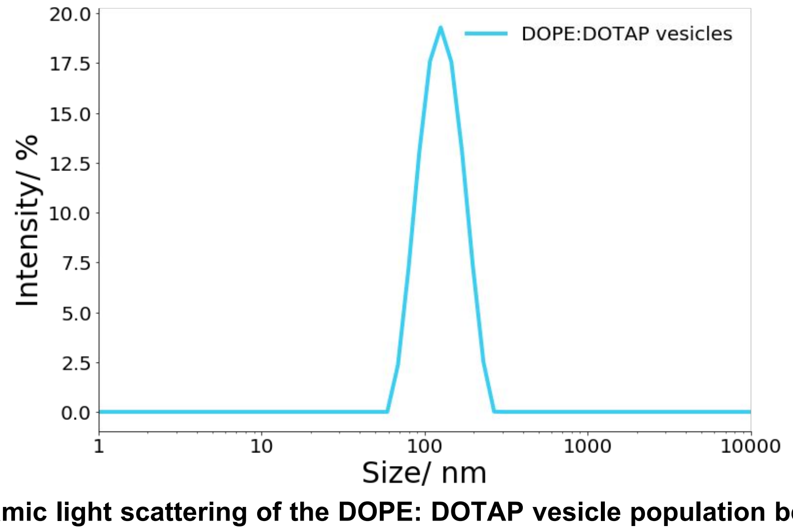

Figure S3: Dynamic light scattering of the DOPE: DOTAP vesicle population before interfacing with the hydrogel artificial cells. The vesicles had an average size of 118 nm with a polydispersity index of 0.07 and were measured by diluting the vesicles in a 1:10 ratio in sucrose buffer (0.5M sucrose, 100mM HEPES, 100mM KCl, 20mM $CaCl_2$ pH 7.4).

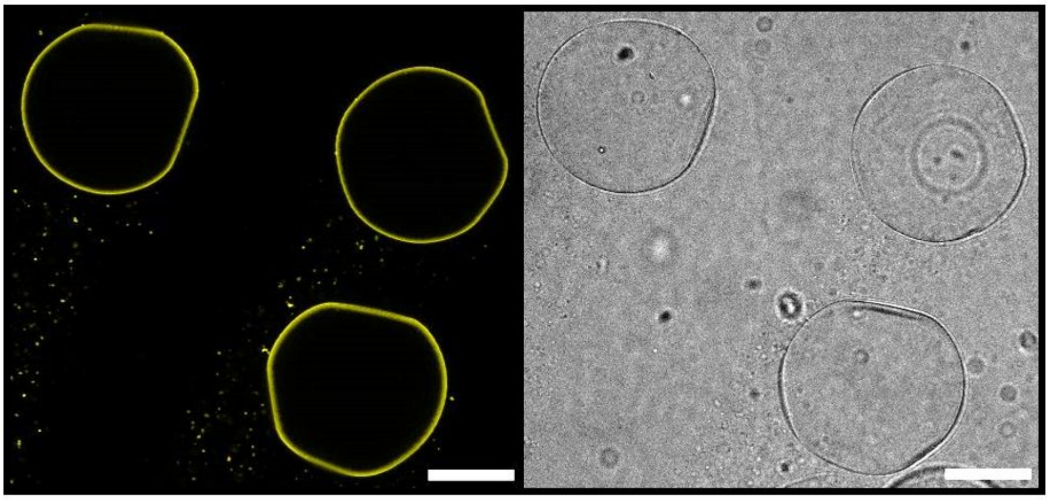

Figure S4: Coating hydrogels with DOPE: DOTAP vesicles. Fluorescence and brightfield images of hydrogels being coated with DOPE: DOTAP vesicles. The vesicles form a membrane on the edge of the hydrogels. The scale bars are 50 µm.

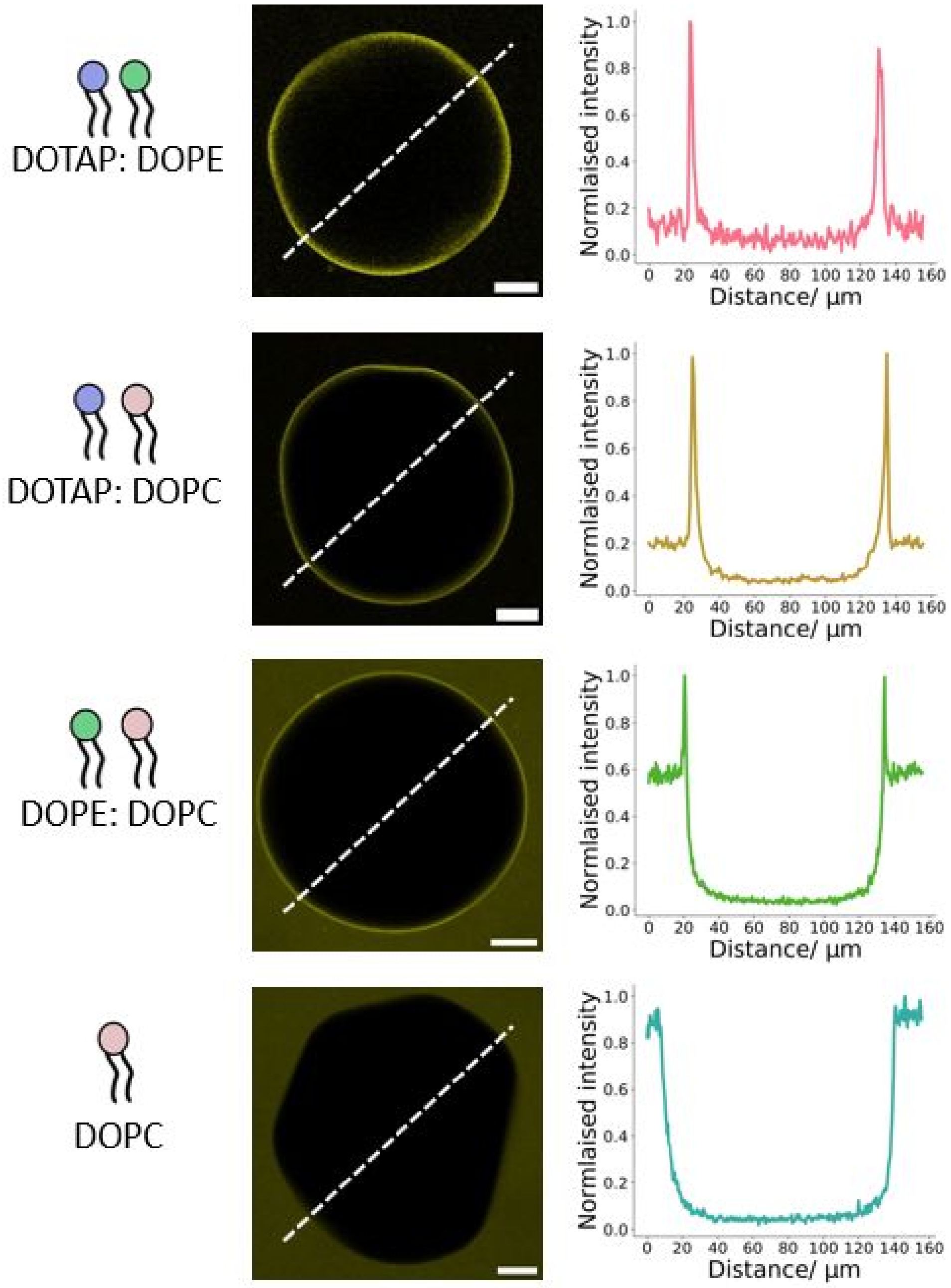

Figure S5: Line profiles of hydrogel artificial cells coated with different lipid compositions. Fluorescence confocal microscopy images of hydrogel artificial cells in different lipid vesicle compositions with associated line profiles. The acquired line profiles are the dotted lines on each image and the corresponding line profile graph is next to each fluorescence image. Upon increasing the fusogencity (DOPE) and charge (DOTAP) the degree of localisation to the hydrogel artificial cell increases. All scale bars are 20 µm.

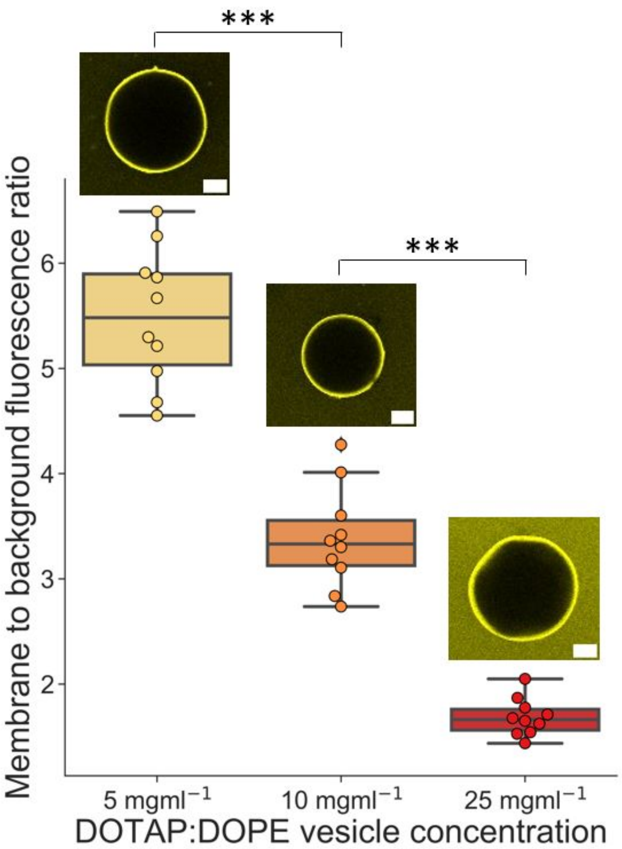

Figure S6: Comparing the membrane prominence at high DOTAP: DOPE concentrations. A box plot with accompanying representative fluorescence confocal images of hydrogel artificial cells in different concentrations of DOTAP: DOPE vesicles with. Above 5 mgml^{-1} the prominence of the membrane signal compared to the background decreased, indicating saturation of the hydrogel with vesicles at the higher concentrations. The box plots were produced from analysing n=10 hydrogel artificial cells for each condition. The scale bars are 20 µm.

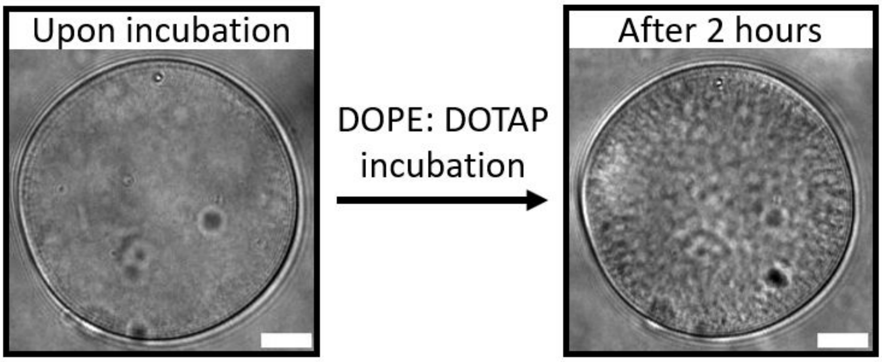

Figure S7: Impact of extended incubation on the hydrogel artificial cells optical texture. Brightfield microscopy images of coated hydrogels at 0 and 2 hours respectively. As the penetration depth of the lipid coating increases, the optical texture also changes, further demonstrating that the lipid structure is penetrating through the hydrogel. The scale bars are 20 µm.

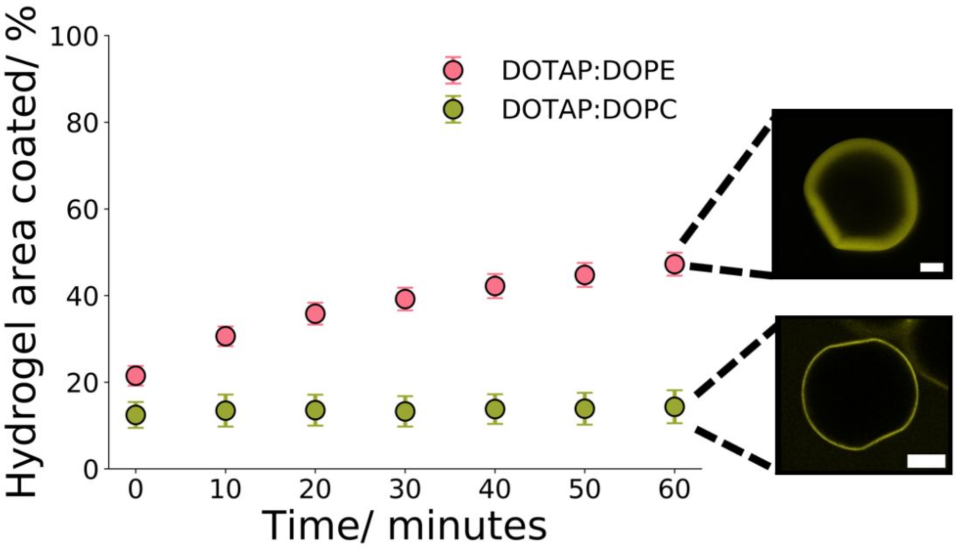

Figure S8: Comparing the impact of lipid vesicle composition on hydrogel artificial cell coating. A plot with accompanying confocal microscopy images after 1 hour demonstrating that the DOTAP: DOPE vesicle composition forms coatings on the hydrogel artificial cells which increase in size over time and penetrate into the hydrogel, while DOTAP: DOPC vesicles only form a coating on the hydrogel interface which remains a constant size over time. The microscopy image scale bars are 20 µm and the error bars represent that standard deviation from n=10 hydrogel artificial cells analysed for each condition.

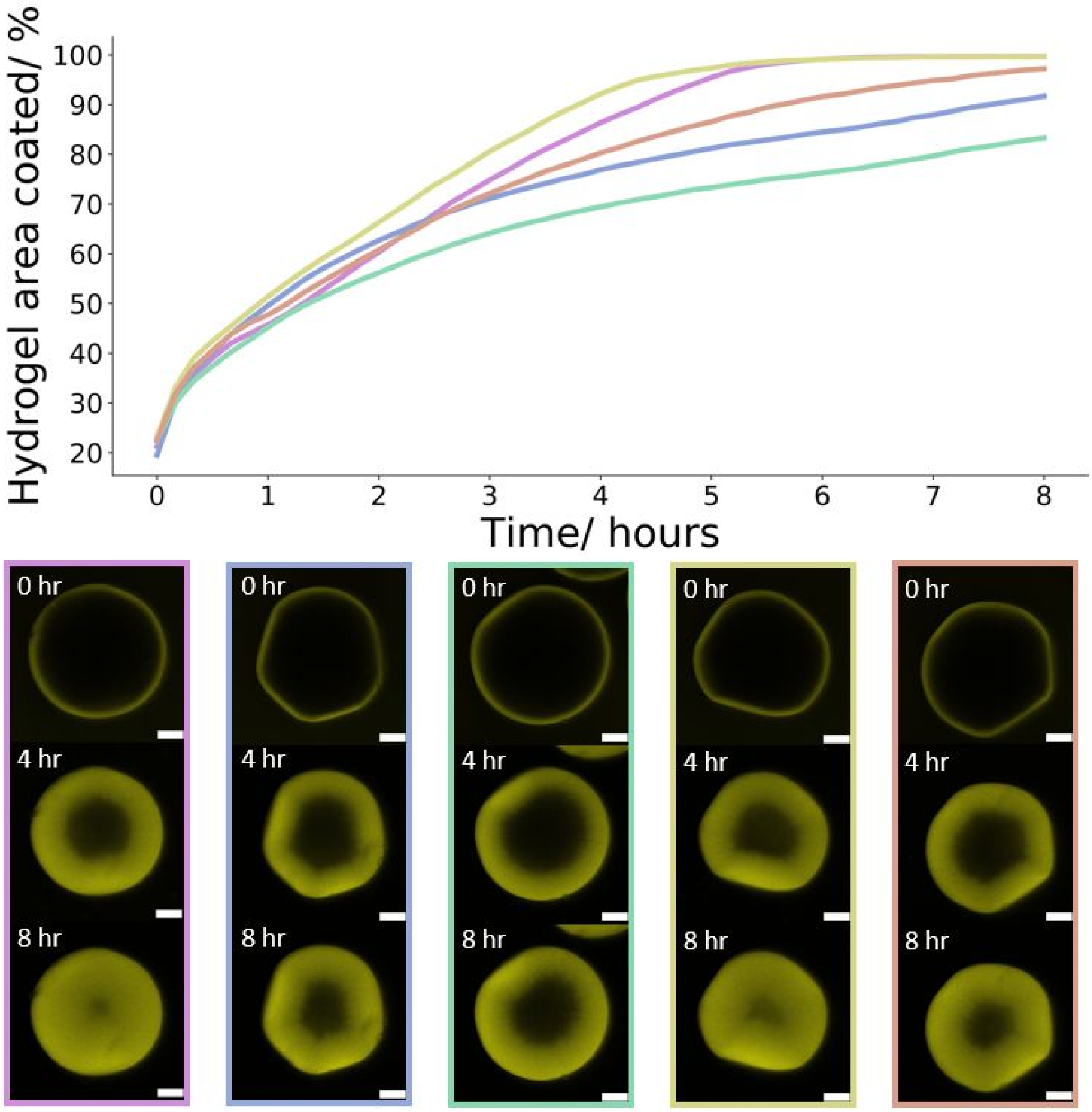

Figure S9: Kinetics of hydrogel artificial cell membrane coating. A graph with accompanying confocal microscopy images showing the development of the lipid membrane coating that penetrates into the hydrogel on 5 different hydrogel artificial cells. The colour of line on the graph corresponds to the series of hydrogel images in the same-coloured box below. After the initial couple of hours, a variation in the degree of hydrogel area coated is observed. We attribute this to the different morphologies of the hydrogel artificial cells. The scale bars are all 20 μm.

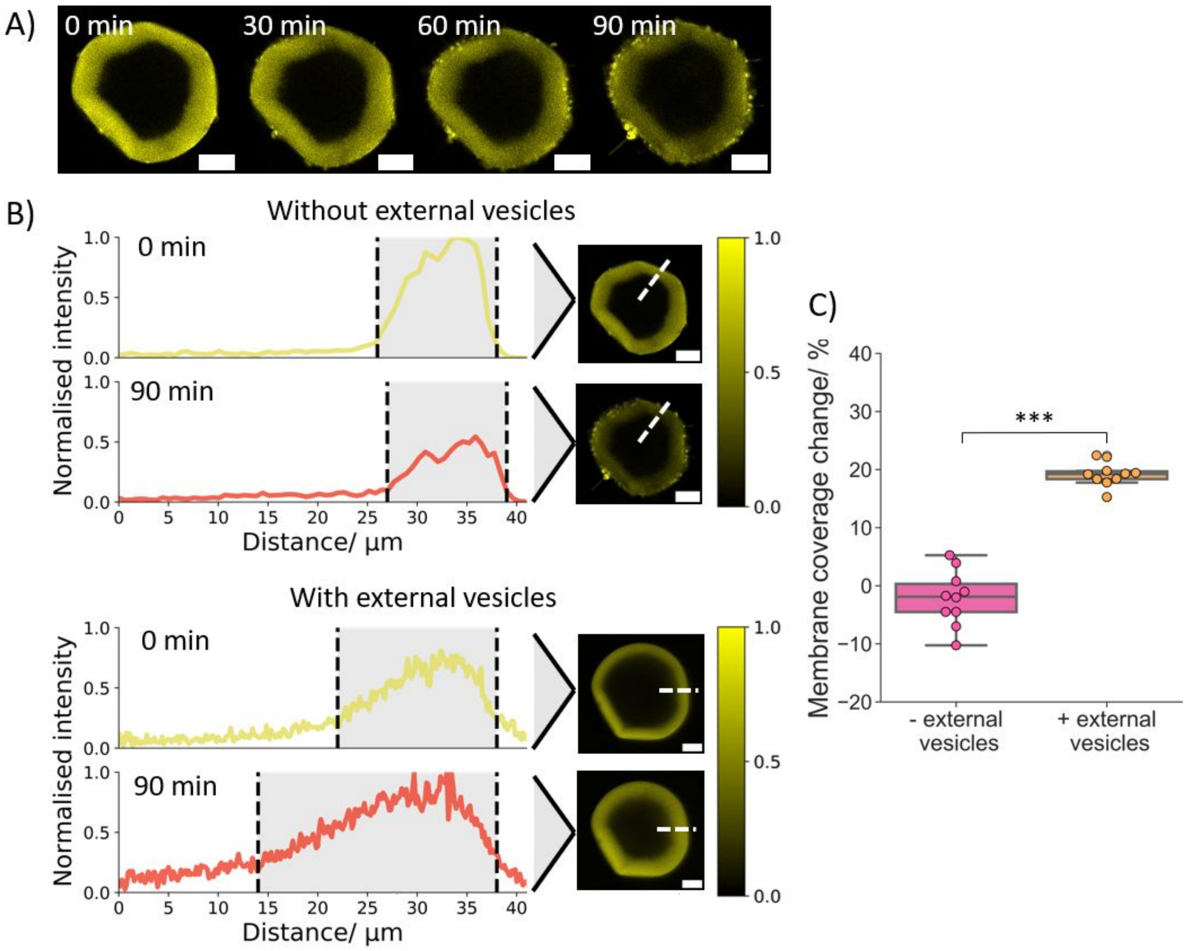

Figure S10: Stability of the membranous hydrogel coating without vesicles in the external solution. A) Fluorescence confocal images over 90 minutes of a membranous hydrogel artificial cell coated for 1 hour previously and left in buffer without any vesicles. Over the 90 minutes the membrane segment can be seen to remain in the same position although it decreases in fluorescence intensity. **B)** Line profile graphs with corresponding confocal microscopy images comparing how the membranous region changes with and without external vesicles over 90 minutes. The hydrogels were coated for 1 hour previously to the time series. The dotted lines on the images represent the line profiles taken for the graphs and the shaded regions on the graphs show the size of the membrane coating. Over 90 minutes with external vesicles the membrane coating penetrates further into the hydrogel and fluorescence intensity while without external vesicles the coating remains a constant size and decreases in fluorescence intensity. The results demonstrate without external vesicles the membrane coating remains a defined size, showing that the penetration depth of the membranous coating can be controlled by altering the incubation time is the surrounding vesicle solution. **C)** Box plots confirming that without external vesicles, the degree of membrane coverage on the hydrogel artificial cells only changes by a small amount (-2.1 ± 4.5 %) over 90 minutes compared to the coated hydrogel artificial cells in the presence of vesicles (19.2 ± 2.0 %) over 90 minutes. The box plots were produced from analysing n=10 hydrogel artificial cells for each condition. The microscopy image scale bars are all 20 µm.

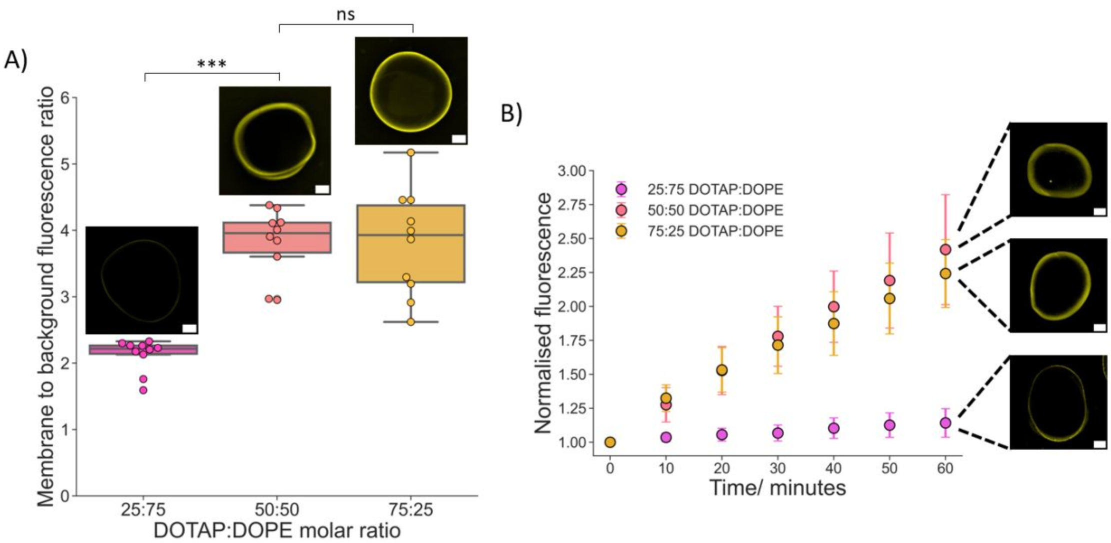

Figure S11: Varying the molar ratio between DOTAP and DOPE. A) Box plot with accompanying microscopy images of hydrogel artificial cells upon addition of DOTAP: DOPE vesicles labelled with DiI. For each condition, the molar ratio of DOTAP: DOPE was altered. It was seen that a more prominent membrane coating was produced when the molar ratio of DOTAP was 50% or larger. **B)** A plot with accompanying microscopy images after 1 hour demonstrating that the DOTAP: DOPE vesicle composition forms coatings on the hydrogel artificial cells which penetrate and increase in size over time with the compositions 50:50 and 75:25 DOTAP: DOPE, no significant difference in coating kinetics was also observed between these compositions. The 25:75 DOTAP: DOPE composition only produced a coating on the hydrogel interface which remained a constant size over time. These results demonstrated that a significant amount of DOTAP (above 50%) was required to produce a DOTAP: DOPE membrane coating that penetrated into the hydrogel artificial cells. All scale bars are 20 μm. The box plots and error bars were produced from analysing n=10 hydrogel artificial cells for each condition.

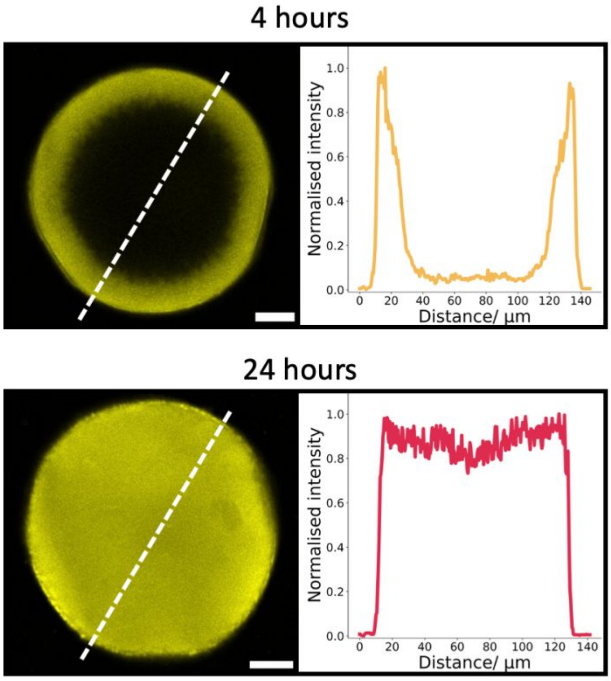

Figure S12: Line profiles of hydrogel artificial cells with a penetrative membrane coating of DOPE: DOTAP vesicles. Fluorescence confocal microscopy images of coated hydrogels at 4 and 24 hours with acquired line profiles from the dotted lines, the corresponding line profile graph is next to each fluorescence image. The line profiles show that upon extended incubation the coating penetration depth increases up to a maximum point where the entire hydrogel artificial cell is coated by a homogenous membrane. All scale bars are 20 μm.

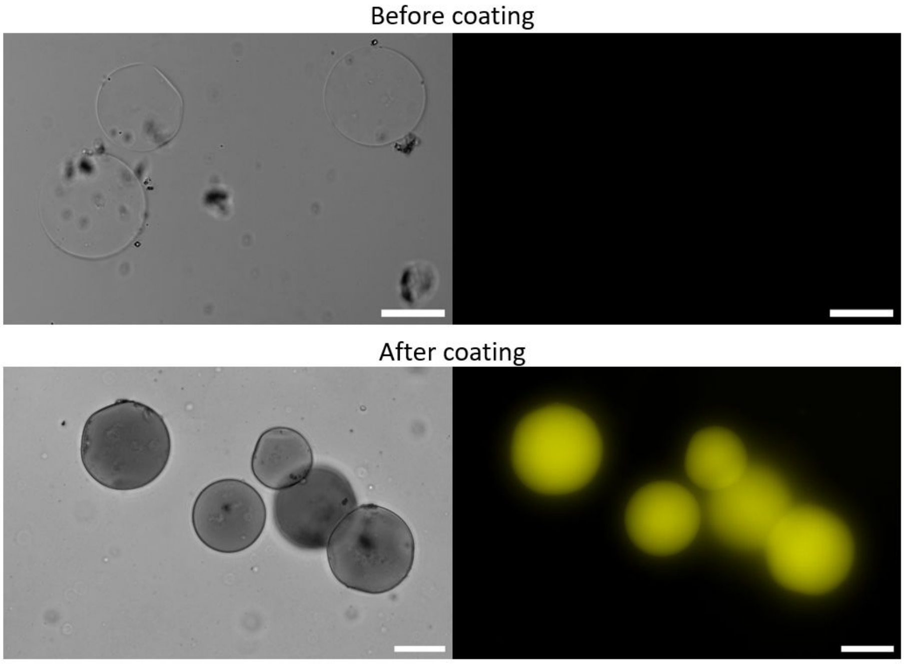

Figure S13: Comparing the optical texture of hydrogel artificial cells before and after 24 hours of coating. Brightfield and fluorescence images of hydrogels before and after membrane coating application, the membrane coating is shown by the yellow fluorescence channel. On applying the coating, the optical texture of the hydrogel artificial cells darkens, showing a change in hydrogel properties. The scale bars are 50 μm.

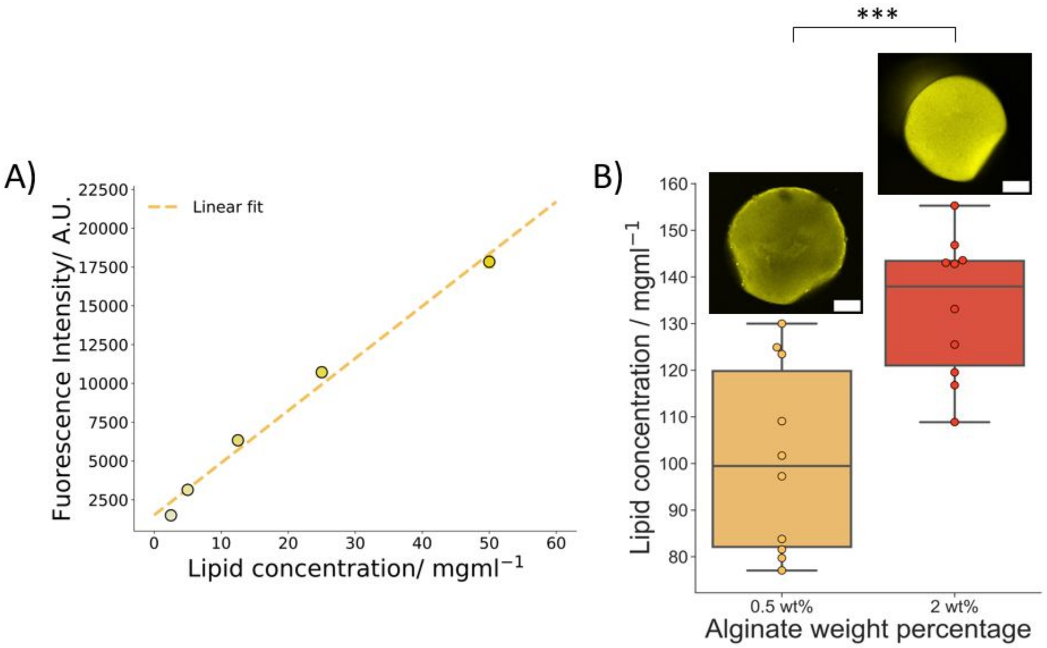

Figure S14: Calculating the lipid concentration on different wt% fully coated hydrogel artificial cells. A) A concentration calibration curve of DOTAP: DOPE vesicles containing Rhodamine dye. On increasing the concentration of the lipid vesicles, the fluorescence intensity also increased. A linear fit was fitted to this relationship (R^2 value = 0.99) The error bars which are the size of the data points were calculated from the standard deviation of n=3 microscopy images of each vesicle concentration. **B)** A box plot with accompanying representative fluorescence confocal images of different weight percentage hydrogel artificial cells with a full membrane coating. The lower weight percentage hydrogel artificial cells possessed a lower concentration of attached lipids. This is attributed to a smaller internal surface area due to the lower concentration of alginate. The box plots were produced from analysing n=10 hydrogel artificial cells for each condition. The scale bars are 20 μm.

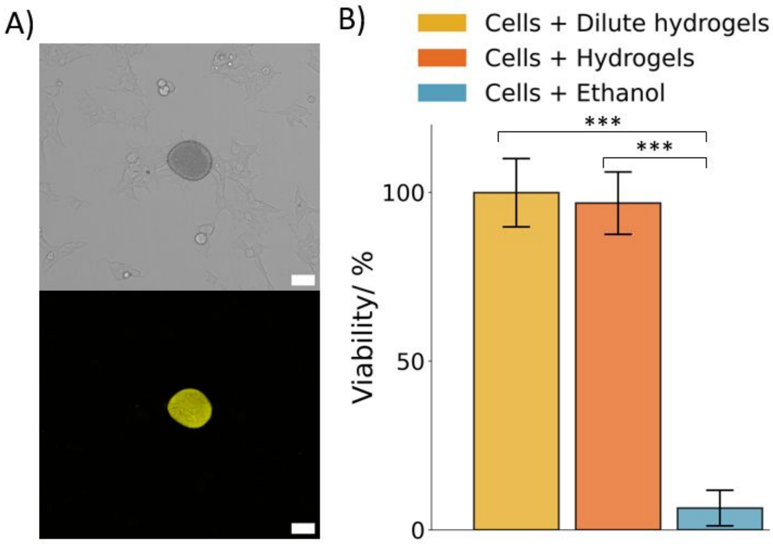

Figure S15: Viability of mammalian cells with coated hydrogel artificial cells. A) Brightfield and fluorescence microscopy images of a hydrogel artificial cell with HEK 293 cells after overnight co-culture. The coated hydrogel artificial cells (labelled with DiI) remained intact in the cell media and the cells possessed no fluorescent signal in comparison to the coated hydrogel artificial cell, indicating that the membrane coating remained on the hydrogel artificial cell. All scale bars are 50 µm. **B)** MTT assay testing the viability of HEK 293 cells co-cultured with hydrogel artificial cells overnight. It could be seen that the cells remained viable in the presence of the hydrogel artificial cells and significantly more viable than cells treated with ethanol. The dilute hydrogel sample possessed approximately 4 hydrogels per well and the other hydrogel sample contained approximately 12 hydrogels per well (~1 or 3% of the well area. n=5 independent wells were analysed for each condition and the mean ± standard deviation was plotted.

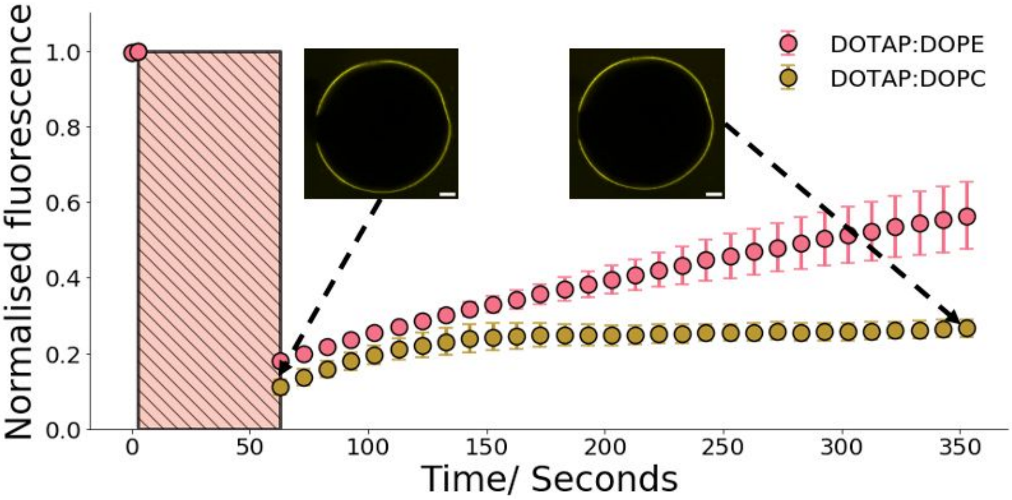

Figure S16: The impact of coating composition on fluorescence recovery. A graph demonstrating the fluorescence recovery of hydrogel artificial cells with a newly applied coating made of DOTAP: DOPE and DOTAP: DOPC. The embedded confocal images demonstrate the recovery of the DOTAP: DOPC hydrogel artificial cell. It can be seen that through using a less fusogenic lipid (DOPC) within the vesicle mixture a significant alteration in fluorescence recovery is observed, the limited recovery is due to the diffusion of new non bleached vesicles towards the hydrogel artificial cells instead of fusion onto the gel surface. The scale bars are 20 µm and the error bars are the standard deviation of n=3 FRAP measurements.

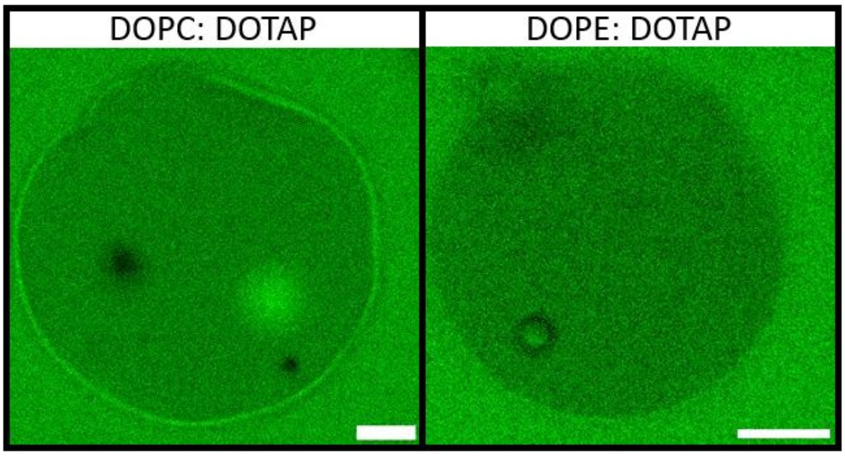

Figure S17: Comparing vesicle bursting. 4 kDa fluorescent Dextran was encapsulated within 100 nm DOPC: DOTAP or DOPE: DOTAP vesicles and incubated within the hydrogels for 1 hour at a concentration of 5 $mgml^{-1}$. The DOPC: DOTAP composition presented a faint ring around the hydrogels after 1 hour indicating vesicle adherence. Meanwhile the DOPE: DOTAP composition lacked this faint ring around the hydrogels, indicating vesicles of this composition burst upon interfacing with the hydrogels. All scale bars are 10 µm.

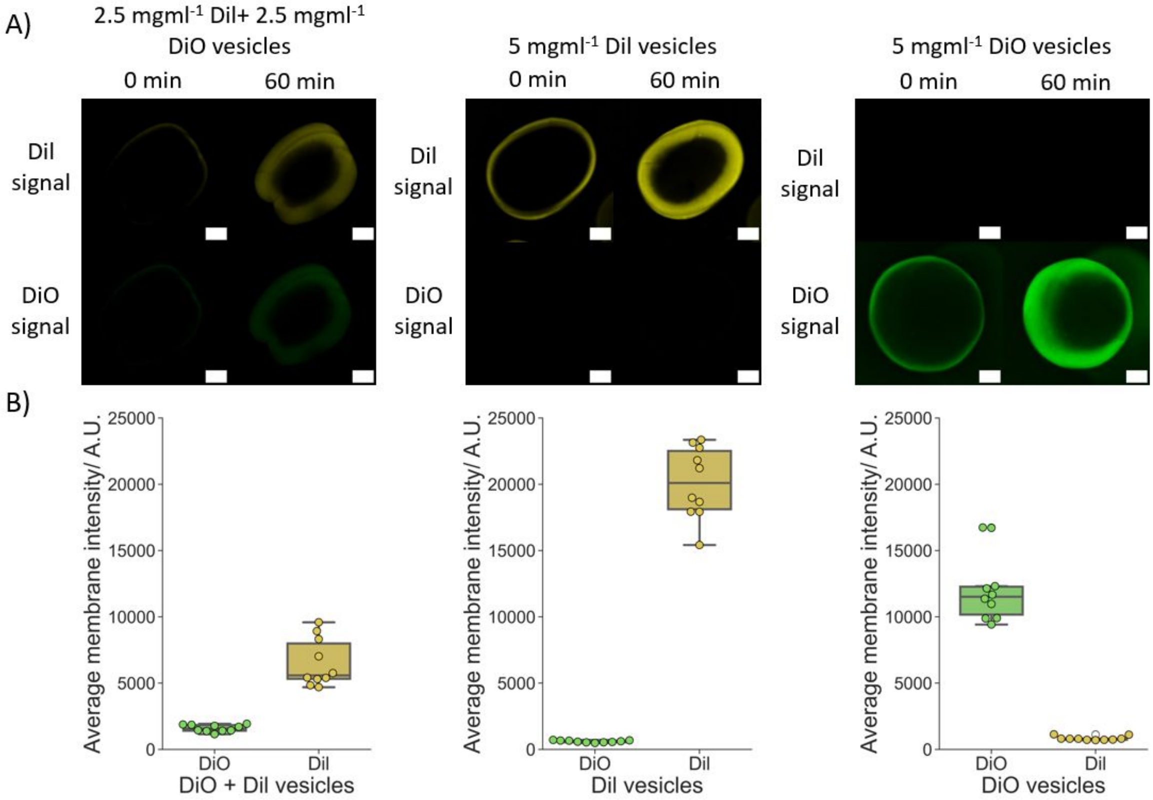

Figure S18: Confirming vesicle fusion on the hydrogel artificial cells. A) Fluorescence microscopy images of hydrogel artificial cells incubated with DOTAP: DOPE vesicle populations containing a Dil dye, a DiO dye or a combination of the two vesicle populations. Images were taken immediately upon vesicle addition and after 1 hour. It can be seen that the hydrogel artificial cells incubated with Dil vesicles or DiO vesicles had only 1 fluorescent signal present on the membranous coating. Meanwhile, the hydrogel artificial cells incubated with a combination of the two vesicle populations, had both a DiO and Dil signal present on the membranous coating. The fluorescent signals from the dyes were also evenly distributed throughout the membranous coating, indicating both vesicle compositions were part of the contiguous membrane. All scale bars are 20 μm. **B)** Box plots analysing the intensity of the membranous coatings after 1 hour of incubation. It can be seen that the hydrogel artificial cells incubated with both vesicle populations had a fluorescent signal from both dyes present on the membranous coating and at a lower fluorescence intensity than the hydrogel artificial cells incubated with just one vesicle population. This indicates that the membranous coating is comprised of the total composition of the external vesicles. Overall, these results demonstrate that vesicles fuse together on the hydrogel interface to create a contiguous membrane comprised of all the vesicles that can coat the hydrogel artificial cells. The box plots were produced from analysing n=10 hydrogel artificial cells for each condition.

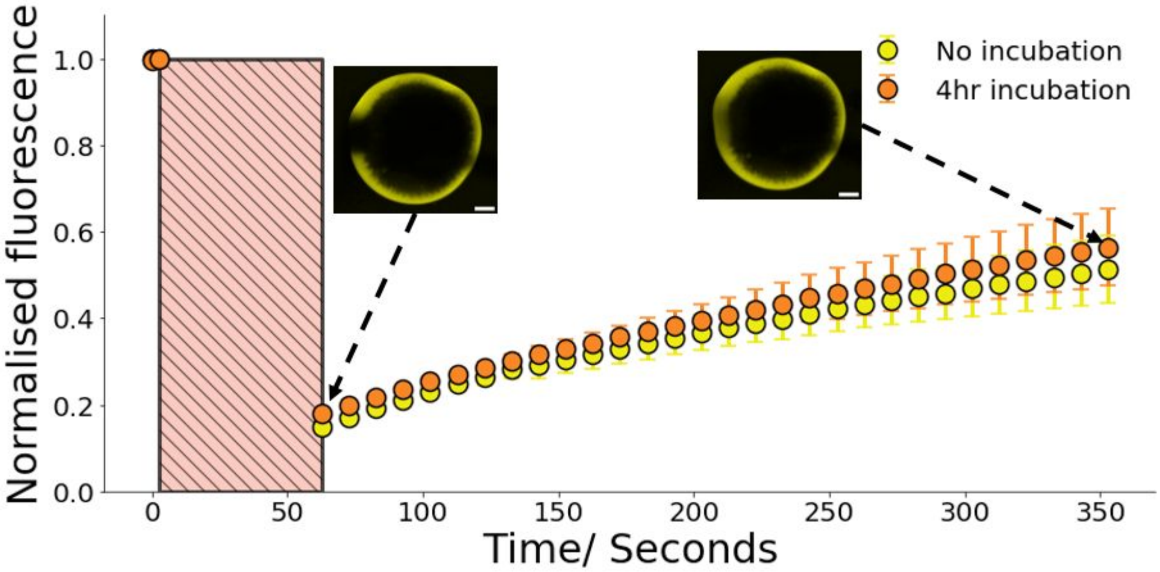

Figure S19: The impact of coating penetration depth upon fluorescence recovery. A graph demonstrating the fluorescence recovery of hydrogel artificial cells with a newly applied coating (no incubation) and a 4hr incubated coating. The embedded confocal images demonstrate the recovery of the 4hr incubated hydrogel artificial cell. This figure shows that the fluorescence recovery time is independent of the coating penetration depth and further verifies that the coating is one continuous structure with the same properties throughout. The scale bars are 20 μm and the error bars are the standard deviation of n=3 FRAP measurements.

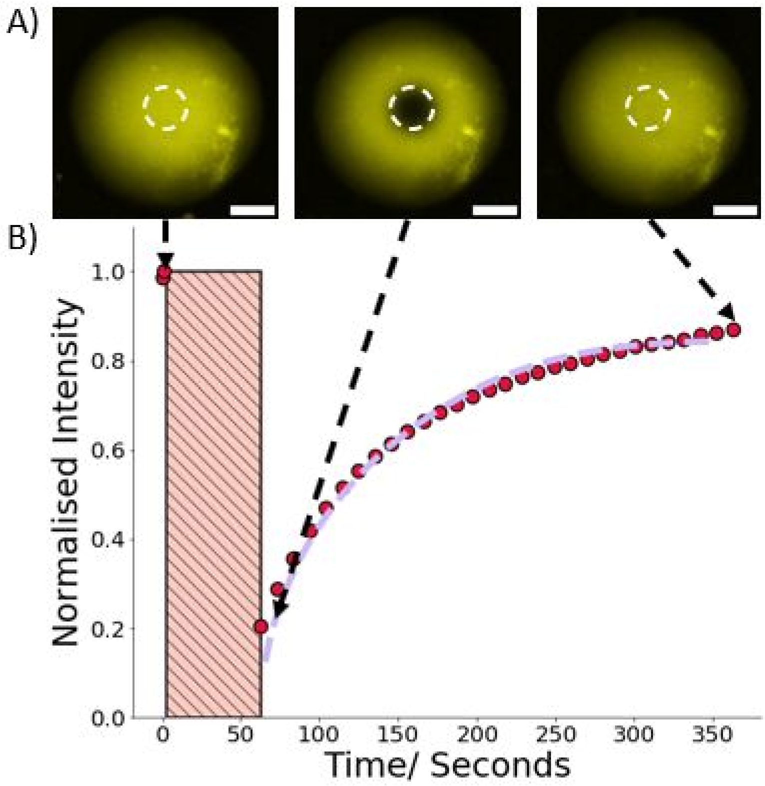

Figure S20: Fitting diffusion coefficients. A) Confocal images showing a fully coated hydrogel artificial cell pulp before, immediately after and 5 minutes after photobleaching. **B)** A graph showing fluorescence recovery upon photobleaching a pulp of a fully coated hydrogel artificial cell, the shaded region indicates the period where the bleaching was occurring, and the dotted blue line represents the fitting of the fluorescence recovery to the Soumpasis equation. The scale bars in section A are 20 µm.

Through bleaching the pulp of a fully coated hydrogel artificial cell with a circular region of interest the fluorescence recovery may be fitted to the theoretical Soumpasis equation[16] where $\tau_D$is the characteristic recovery time, *A* is the recovery level of the fluorescence, *t* is the time and J_0 and $J_1$are modified Bessel functions of the first kind (**Equation S1**).

$$F(t) = Ae^{(-\frac{2\tau_D}{t})}\left[J_0\left(\frac{2\tau_D}{t}\right) + J_1\left(\frac{2\tau_D}{t}\right)\right]$$

(Equation S1)

From extracting the characteristic recovery time equation S2 may be utilised to find the diffusion coefficient with knowledge of ω, the bleach region radius.

$$D = \frac{\omega^2}{4\tau_D}$$

(Equation S2)

Through fitting the Soumpasis equation to the fluorescence recovery the diffusion coefficient obtained is 0.045 $\mu m^2 s^{-1}$. When compared to a GUV the diffusion coefficient is significantly smaller (3.7 $\mu m^2 s^{-1}$ for a GUV[17]). However, this model does not account for the 3-dimensional hydrogel network upon which the lipid coating is on which will reduce the diffusion coefficient as the fluorophore recovery pathway will not be linear.

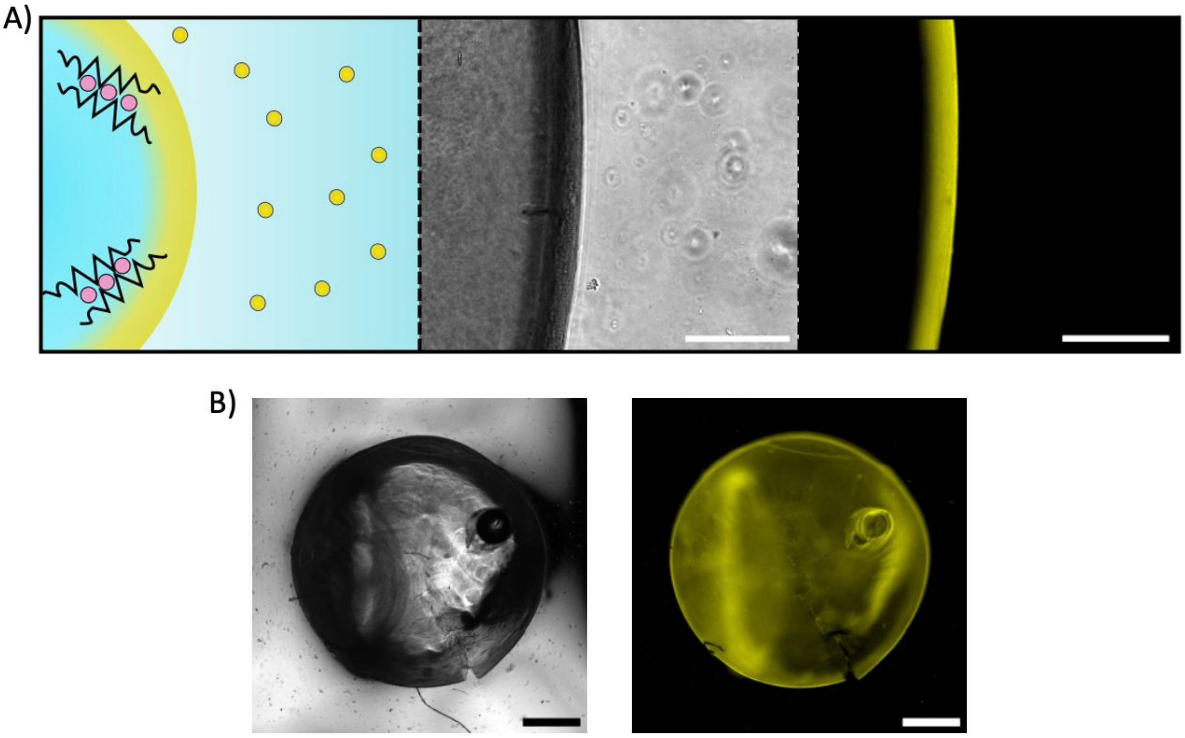

Figure S21: Coating of millimetre sized hydrogels. A) A schematic, brightfield image and fluorescence image of a millimetre sized hydrogel coated with DOTAP: DOPE vesicles. A localisation of fluorescence signal can again be seen at the hydrogel/ solution interface accompanied by a change in brightfield optical texture. This indicates that the same mechanism is patterning gels of this size and the mechanism for patterning can be readily applied to different size alginate hydrogels. The scale bars are 100 µm. **B)** Brightfield and fluorescence images of the entire coated hydrogel, this example is ~20 times larger than the hydrogel artificial cells. The scale bar is 500 µm.

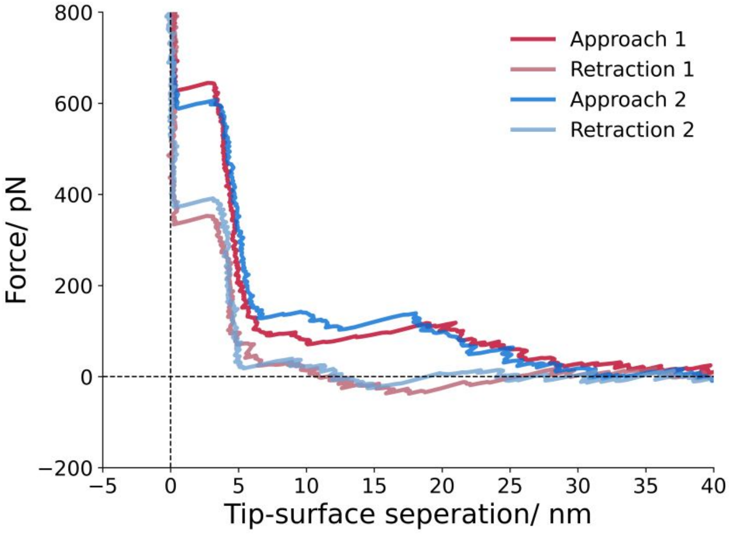

Figure S22: AFM force separation curve on mica coated with DOTAP: DOPE vesicles. 2 force distance curves are shown with the approach and retraction portions shaded differently. The Individual bilayers are 5.5 ± 0.3 nm in depth with a typical penetration force of the bilayer next to the surface being 600pN. Two further bilayers on top of the first bilayer can be clearly discriminated, but more loosely bound bilayers are on top, with a total of 6 bilayers, these are shown through the multiple sharp changes in the curve direction. Additionally, the retract curve is expelled from bilayer at a positive force, indicating the positive pressure on the tip which is a function of bilayer fluidity. This matches the appearance of the coated hydrogel artificial cell force separation curves, confirming that a multilamellar lipid coating is present on the hydrogel artificial cells.

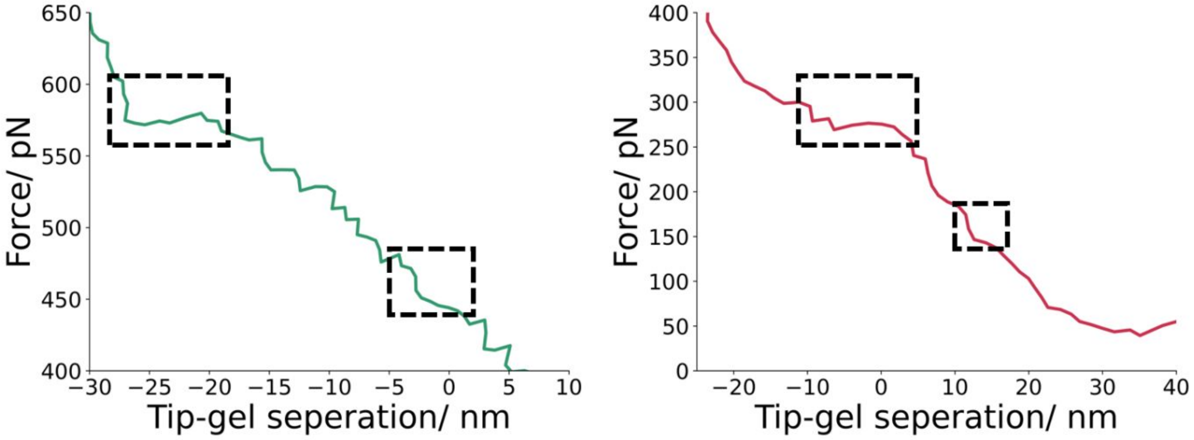

Figure S23: Sections of AFM force separation curves of hydrogel artificial cells coated with DOTAP: DOPE vesicles. Within all of the force separation curves a sudden change in curve direction, is present (with some of the most obvious events shown by the dotted boxes), indicative of the AFM tip breaking through a lipid bilayer[18]. As there are multiple of these events, the membranous coating is comprised of multiple layers.

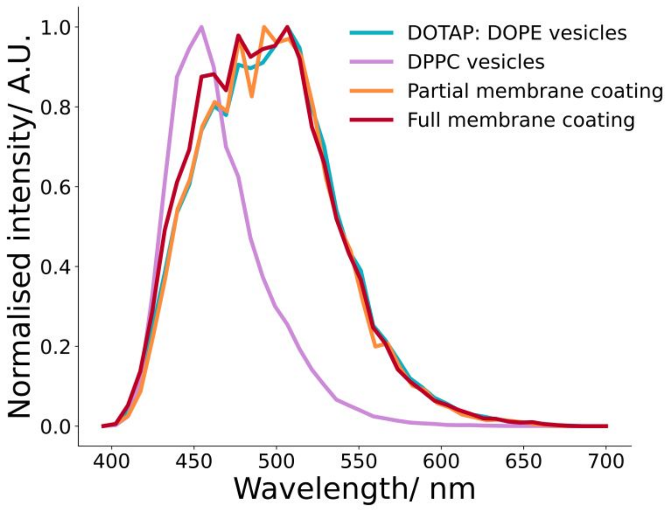

Figure S24: Comparing the effect of the coating on the emission spectra of Laurdan. A graph showing the emission spectra of Laurdan dye in DOTAP: DOPE vesicles, DPPC vesicles and within different membrane coating sizes on the hydrogel artificial cells. The emission spectra of all three DOTAP: DOPE membrane environments containing Laurdan is similar, showing that on membrane application to hydrogels, the membrane properties of the vesicle composition are conserved. Furthermore, the DOTAP: DOPE vesicle properties are different from the gel phase DPPC vesicles, indicating that the coating is fluidic.

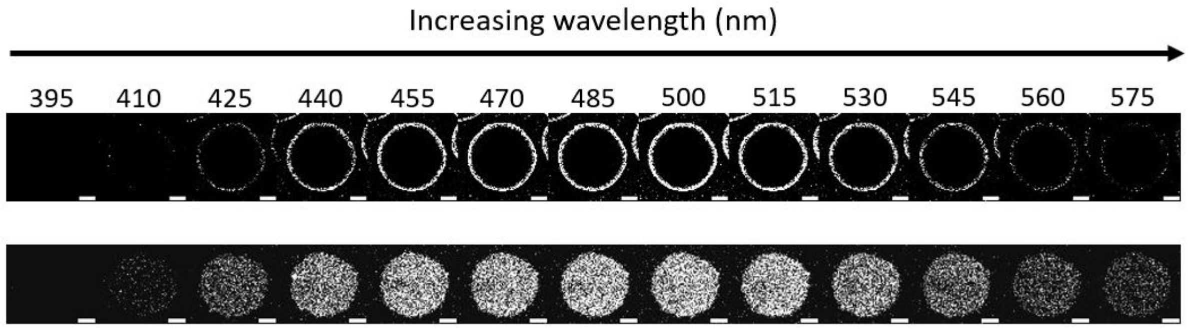

Figure S25: Fluorescence images of partial and fully coated hydrogel artificial cells at a variety of different wavelengths. Representative confocal microscopy images of the emission spectra of Laurdan dye on partial and fully coated hydrogel artificial cells. The fluorescence intensity is at a maximum around 500 nm in both examples. The scale bars are 20 µm.

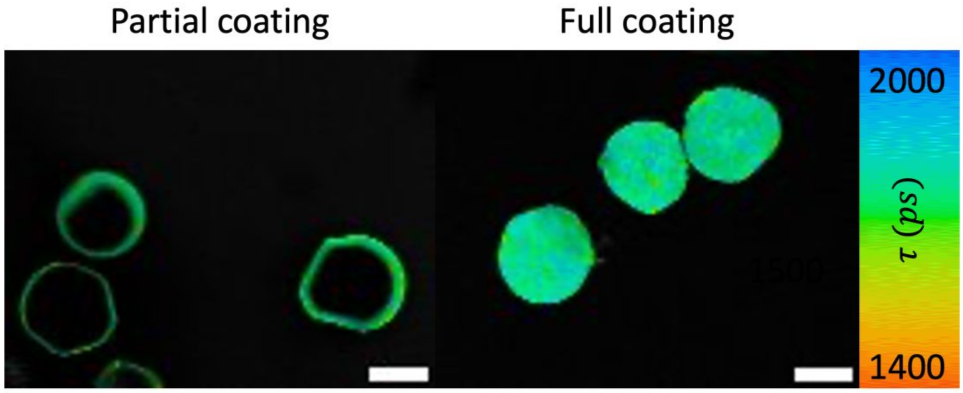

Figure S26: Representative FLIM images of partial and fully coated hydrogel artificial cells. The images show the lifetime of the fluorescent BODIPY dye present in the membrane coating on partially and fully coated hydrogel artificial cells. A minimal difference in lifetime could be seen between the two populations. The scale bars are 50 µm.

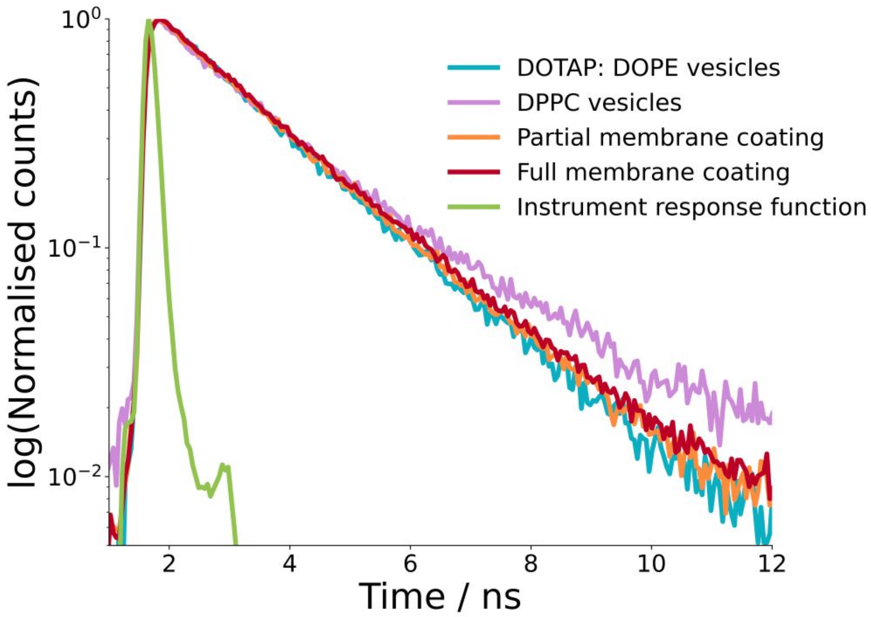

Figure S27: Fluorescence decay traces of the coated hydrogel artificial cells and DOTAP: DOPE vesicles. A graph showing example time-resolved fluorescence decay traces of BODIPY from DOTAP: DOPE vesicles, DPPC vesicles and membrane coated hydrogel artificial cells. The instrument response function is also shown. Between all the DOTAP: DOPE samples little change in decay is seen, indicating the membrane properties of the vesicle are conserved in the membranous hydrogel artificial cell coating. Furthermore, the DOTAP: DOPE vesicles have a different decay trace to the gel phase DPPC vesicles, indicating that the coating is fluidic.

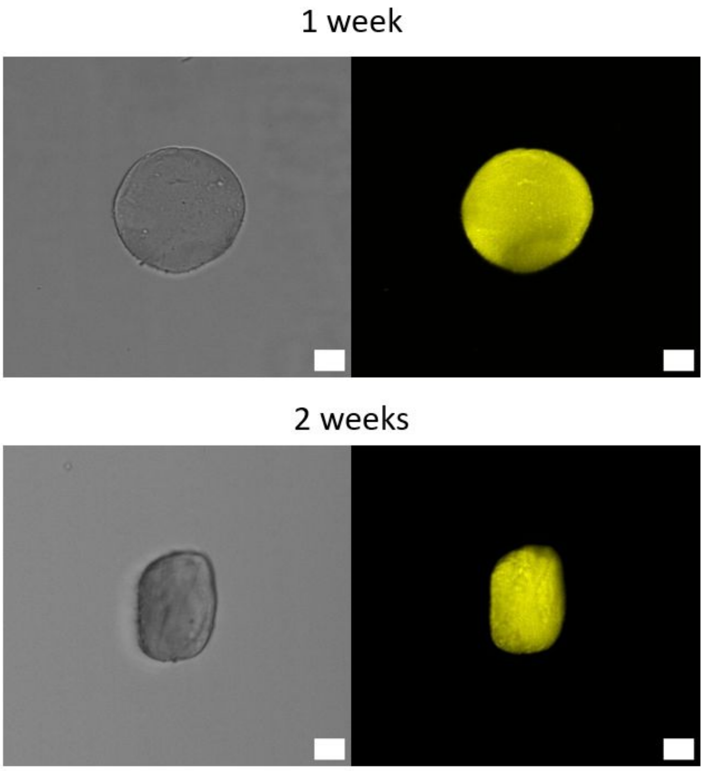

Figure S28: Stability of coated hydrogel artificial cells over time. Fluorescence and brightfield microscopy images of hydrogel artificial cells after 1 and 2 weeks. The hydrogel artificial cells were coated with DOTAP: DOPE vesicles labelled with DiI dye for 24 hours before being resuspended in fresh sucrose buffer without any vesicles. The coated hydrogel artificial cells were imaged after 1 and 2 weeks where it was observed that the coated hydrogel artificial cells were stable and remained coated over these periods of time. All scale bars are 20 μm.

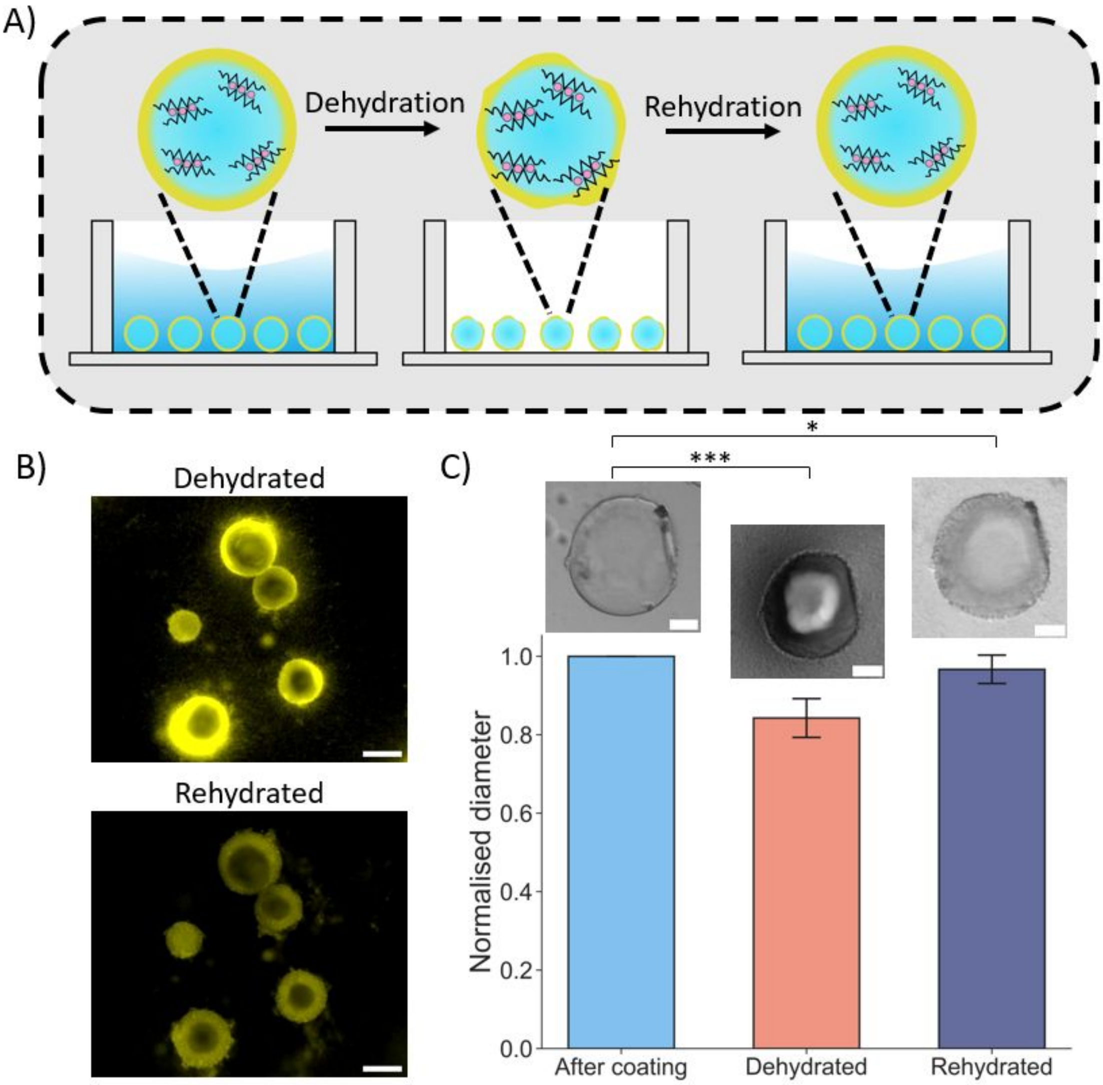

Figure S29: Dehydration and rehydration of coated hydrogel artificial cells. A) A schematic demonstrating the process used to dry and rehydrate the membrane coated hydrogel artificial cells. Membrane coated hydrogel artificial cells were dried in buffer for 2 hours at 60 °C before being rehydrated in the same buffer. **B)** Fluorescence microscopy images of a population of hydrogel artificial cells before and after rehydration. The membrane coating remains on the hydrogels after dehydration and subsequent rehydration. The scale bars are 50 µm. **C)** A bar chart with associated brightfield images showing the diameter of membrane coated hydrogel artificial cells after dehydration and after the subsequent rehydration. The hydrogels return to 97 % of their original size after rehydration. The error bars represent the standard deviation of n=10 membrane coated hydrogel artificial cells, and the scale bars are 20 µm.

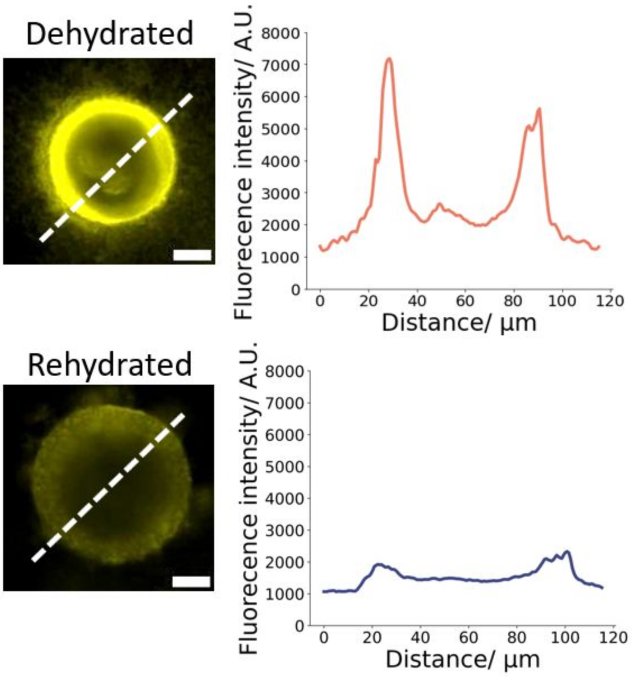

Figure S30: Line profiles of dehydrated and rehydrated coated hydrogel artificial cells. Fluorescence microscopy images of a coated hydrogel when dehydrated and rehydrated with buffer. Acquired line profiles from each image are taken from the dotted lines and the corresponding line profile graph is next to each fluorescence image. The line profiles show that upon rehydration, the fluorescence intensity decreases, indicating the spreading out of the fluorescent membrane over the swollen hydrogel network. All scale bars are 20 µm.

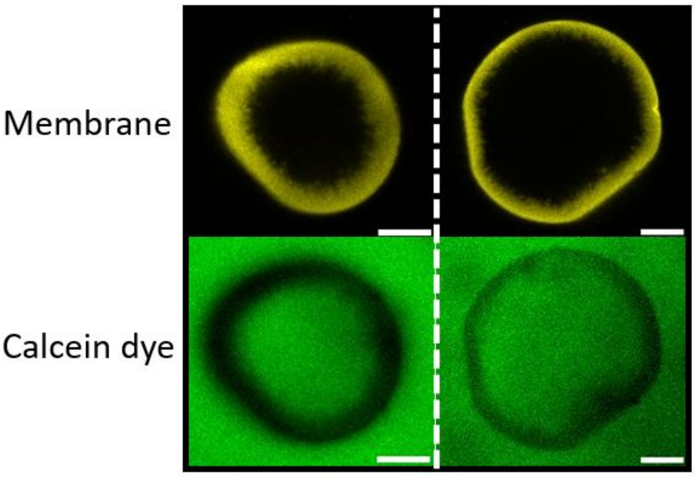

Figure S31: Calcein exclusion in membrane coated hydrogel artificial cell regions. Fluorescence images of membrane coated gels incubated in calcein dye for 30 minutes. There is limited calcein signal in the regions coated by the lipid membrane. The more the coating penetrates into the hydrogel network the stronger the exclusion of calcein dye. This shows that the membrane penetration depth can finely alter the permeability of the gel. The scale bars are 20 µm.

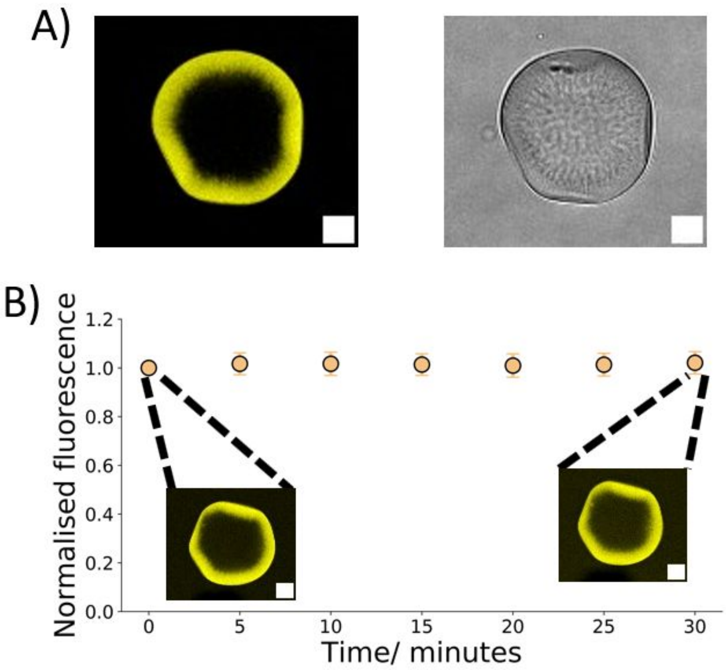

Figure S32: Resorufin permeation in partially coated hydrogel artificial cells. A) Confocal microscopy images of a hydrogel artificial cell coated for 1 hour after the addition of 0.5 mM Resorufin. The Resorufin fluorescence can be seen to be localised in the section of the hydrogel with a membranous coating, showing that Resorufin localises to the hydrogel membrane regardless of coating size. **B)** A plot with accompanying confocal microscopy images demonstrating that Resorufin fully permeates into the membrane on a time scale of seconds and does not enrich further over 30 minutes. The error bars represent that standard deviation from n=10 hydrogel artificial cells analysed. All microscopy image scale bars are 20 µm.

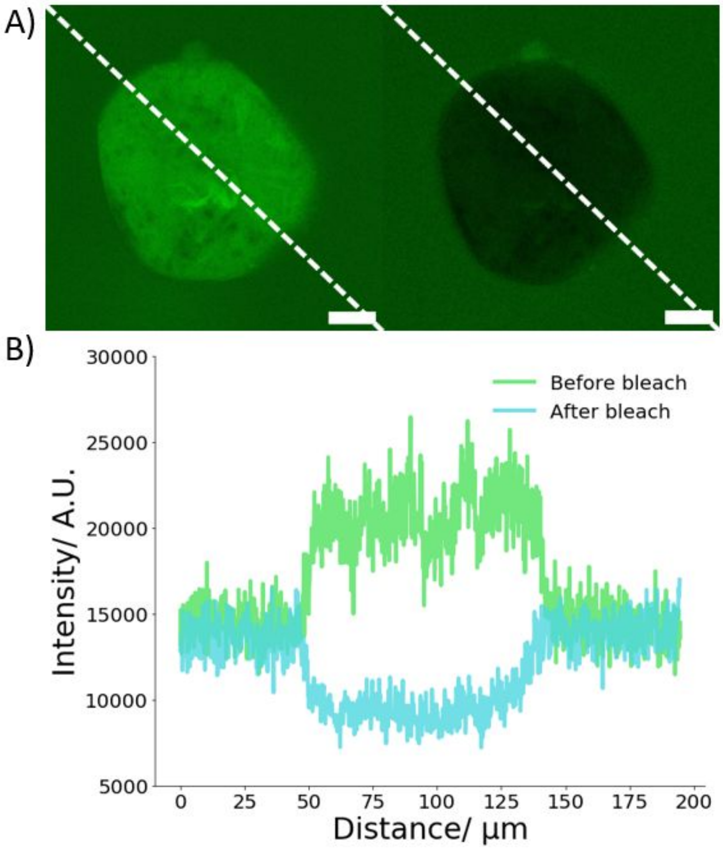

Figure S33: Demonstrating separation between the hydrogel cargo and the exterior solution. Hydrogels coated for 24 hours with DOTAP: DOPE SUVs and immersed in 0.25 mM calcein were diluted by ratio of 1:2 in sucrose buffer (0.5M sucrose, 100mM HEPES, 100mM KCl, 20mM $CaCl_2$ pH 7.4). The gels were then bleached for 10s. It could be seen that the fluorescence intensity of the interior of the gel dropped significantly and below that of the background demonstrating that limited diffusion of calcein dye was occurring between the gel interior and the exterior solution. The scale bars in panel A are 20 µm.

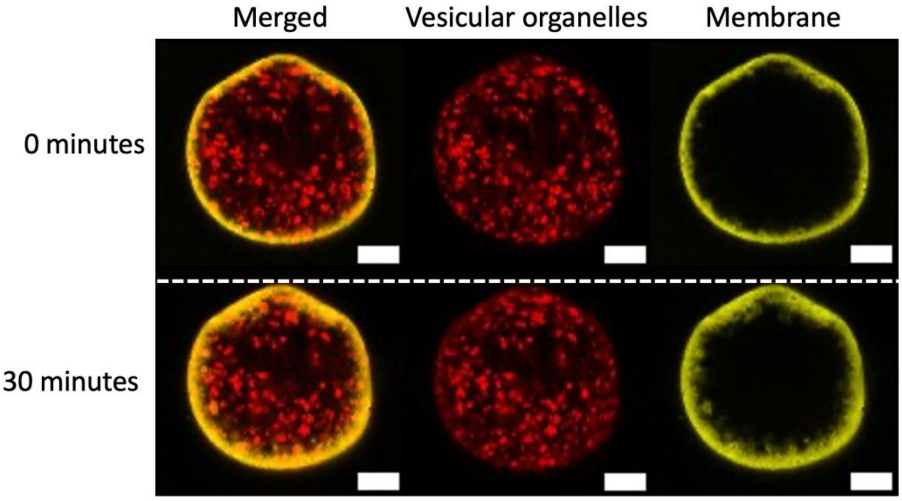

Figure S34: Kinetics of gel coating on hydrogel artificial cells including organelles. The images show vesicle organelles labelled with a cy5 tagged lipid and the membrane labelled with a rhodamine tagged lipid. Over a 30-minute time period the coating penetrated further into the hydrogel network and had little effect on the organelles (shown by their visibility after 30 minutes). This demonstrates that the lipid coating does not interfere with existing organelles structures and can be tuned the same way as with gels that contain no organelles. The scale bars are 20 µm.

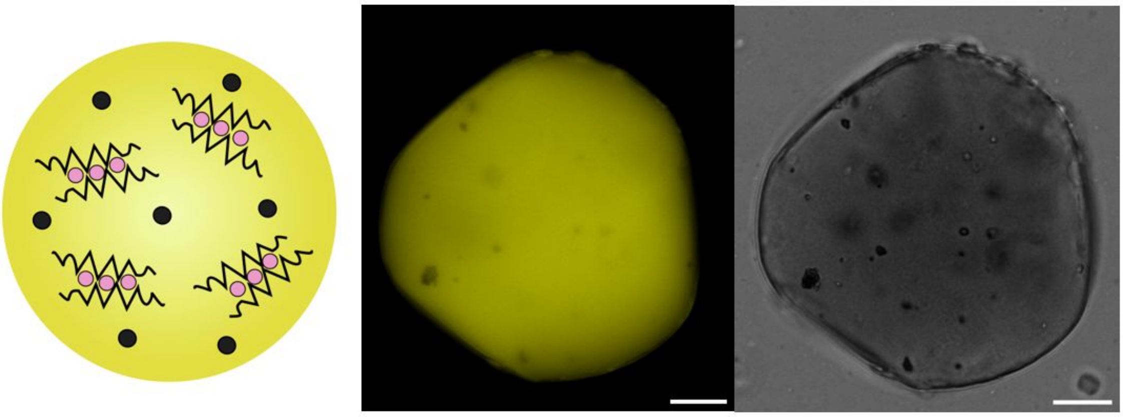

Figure S35: Membrane coating on hydrogel artificial cells with magnetic particle organelles. A schematic with accompanying fluorescence and brightfield images showing the assembly of a membrane coating on a hydrogel artificial cell containing magnetic particle organelles. The fluorescence is from a rhodamine tagged lipid present in the membrane coating. The membrane coating surrounds the magnetic particle organelles (seen in brightfield image), showing that solid organelles may be incorporated within coated hydrogel artificial cells. The scale bars are 20 μm.

Supporting videos

Video S1: Microfluidic production of hydrogels. The scale bar is 200 μm.

Video S2: 8-hour fluorescence timelapse of DOPE: DOTAP SUV incubation with a population of hydrogel artificial cells. The scale bar is 50 μm.

Video S3: 8-hour fluorescence timelapse of DOPE: DOTAP SUV incubation with a single hydrogel artificial cell. The scale bar is 20 μm.

Video S4: Recovery of a DOTAP: DOPE membrane on a hydrogel artificial cell. The scale bar is 20 μm.

Video S5: Recovery of a DOTAP: DOPC membrane on a hydrogel artificial cell. The scale bar is 20 μm.

Video S6: Recovery of a partially penetrated DOTAP: DOPE membrane on a hydrogel artificial cell. The scale bar is 20 μm.

Video S7: Rehydration of membrane coated hydrogel artificial cells. The scale bar is 100 μm.

Video S8: Prevention of calcein permeation into fully coated hydrogel artificial cells. The scale bar is 20 μm.

References

1. Kim, B. K. *et al.* DOTAP/DOPE ratio and cell type determine transfection efficiency with DOTAP-liposomes. *Biochim Biophys Acta Biomembr* **1848**, 1996–2001 (2015).
2. Bloom, M. & Evans, E. Physical properties of the fluid lipid-bilayer component of cell membranes: A perspective. *Q Rev Biophys* **24**, 293–397 (1991).
3. Li, D.-Y., Zhou, Z.-H., Yu, Y.-L. & Deng, N.-N. Microfluidic construction of cytoskeleton-like hydrogel matrix for stabilizing artificial cells. *Chem Eng Sci* **264**, 118186 (2022).
4. Walther, T., Jahnke, K., Abele, T. & Göpfrich, K. Printing and Erasing of DNA-Based Photoresists Inside Synthetic Cells. *Adv Funct Mater* **32**, 2200762 (2022).
5. Saleem, Q., Liu, B., Gradinaru, C. C. & MacDonald, P. M. Lipogels: Single-lipid-bilayer-enclosed hydrogel spheres. *Biomacromolecules* **12**, 2364–2374 (2011).
6. Saeki, D., Honma, K. & Okumura, Y. Preparation of hydrogel-supported giant vesicles via a lipid-coated hydrogel transfer method with electrostatic interaction. *Colloids Surf A Physicochem Eng Asp* **709**, 136074 (2025).
7. Llopis-Lorente, A. *et al.* Artificial cells with viscoadaptive behavior based on hydrogel-loaded giant unilamellar vesicles. *Chem Sci* **15**, 629–638 (2023).
8. Hettiarachchi, K. & Lee, A. P. Polymer-lipid microbubbles for biosensing and the formation of porous structures. *J Colloid Interface Sci* **344**, 521–527 (2010).
9. Wang, M. *et al.* Assembling responsive microgels at responsive lipid membranes. *Proc Natl Acad Sci U S A* **116**, 5442–5450 (2019).
10. Tanaka, A., Nakashima, H., Kashimura, Y. & Sumitomo, K. Electrostatically induced planar lipid membrane formation on a cationic hydrogel array by the fusion of small negatively charged unilamellar vesicles. *Colloids Surf A Physicochem Eng Asp* **477**, 63–69 (2015).
11. Chin, C. L., Huang, L. J., Lu, Z. X., Weng, W. C. & Chao, L. Using the Water Absorption Ability of Dried Hydrogels to Form Hydrogel-Supported Lipid Bilayers. *Gels* **9**, 751 (2023).
12. Rahni, S. & Kazakov, S. Hydrogel Micro-/Nanosphere Coated by a Lipid Bilayer: Preparation and Microscopic Probing. *Gels* **3**, 7 (2017).
13. Versluis, F. *et al.* Negatively Charged Lipid Membranes Catalyze Supramolecular Hydrogel Formation. *J Am Chem Soc* **138**, 8670–8673 (2016).
14. Tam, N. W., Becker, A., Mangiarotti, A., Cipitria, A. & Dimova, R. Extracellular vesicle mobility in collagen I hydrogels is modulated by RGD-binding integrins. *ACS Nano* **18**, (2024).
15. Trantidou, T., Elani, Y., Parsons, E. & Ces, O. Hydrophilic surface modification of pdms for droplet microfluidics using a simple, quick, and robust method via pva deposition. *Microsyst Nanoeng* **3**, 1–9 (2017).
16. Soumpasis, D. M. Theoretical analysis of fluorescence photobleaching recovery experiments. *Biophys J* **41**, 95 (1983).
17. Pincet, F. *et al.* FRAP to characterize molecular diffusion and interaction in various membrane environments. *PLoS One* **11**, (2016).
18. Et-Thakafy, O. *et al.* Mechanical Properties of Membranes Composed of Gel-Phase or Fluid-Phase Phospholipids Probed on Liposomes by Atomic Force Spectroscopy. *Langmuir* **33**, 5117–5126 (2017).
